# Rhinoceromics: a multi-amplicon study with clinical markers to transferrin saturation levels in ex-situ black rhinoceros (*Diceros bicornis michaeli*)

**DOI:** 10.3389/fmicb.2025.1515939

**Published:** 2025-05-29

**Authors:** Linda G. R. Bruins-van Sonsbeek, Martie C. M. Verschuren, Sonja Kaal, Peter W. Lindenburg, Kees (C.) W. Rodenburg, Marcus Clauss, Arjen G. C. L. Speksnijder, Victor P.M.G. Rutten, Bas F. J. Bonnet, Floyd Wittink

**Affiliations:** ^1^Veterinary Department, Rotterdam Blijdorp Zoo, Rotterdam, Netherlands; ^2^Avans University of Applied Sciences, Breda, North Brabant, Netherlands; ^3^LCAB, Department of Analytical BioSciences, University of Applied Sciences Leiden, Leiden, Netherlands; ^4^Clinic for Zoo Animals, Exotic Pets and Wildlife, University of Zurich, Zurich, Switzerland; ^5^LCAB, Department of Environmental Metagenomics, University of Applied Sciences Leiden, Leiden, Netherlands; ^6^Department Understanding Evolution, Naturalis Biodiversity Center, Leiden, Netherlands; ^7^Section Immunology, Div Infectious Diseases and Immunology, Dept Biomolecular Health Sciences|Faculty of Veterinary Medicine, Utrecht University, Utrecht, Netherlands; ^8^Department of Veterinary Tropical Diseases, Faculty of Veterinary Science, University of Pretoria, Pretoria, South Africa; ^9^LCAB, Department of Bioinformatics, University of Applied Sciences Leiden, Leiden, Netherlands

**Keywords:** *Diceros bicornis michaeli*, black rhinoceros, iron overload disorder, short- and medium-chain fatty acid analysis, microbiome, vitamin D, inflammatory markers, mycobiome

## Abstract

Iron overload disorder (IOD) is a common condition in ex-situ black rhinoceroses (*Diceros bicornis*), although it has not been reported in the wild. This study aimed to gain a deeper understanding of the relationship between 25-hydroxy vitamin D [25(OH)D], inflammatory markers, insulin levels, the gut microbiome, dietary components, and transferrin saturation (TS) in *ex-situ* black rhinoceroses. Blood and fecal samples from 11 black rhinoceroses at five different European zoological institutions were monitored over a 1-year period. Inflammatory markers such as interleukin 6 (IL-6), serum amyloid A (SAA), interferon γ (IFN-γ), and tumor necrosis factor α (TNF-α) were analyzed. Our study corroborates the findings of previous research, which demonstrated that insulin, inflammatory markers, and TS% are higher in *ex-situ* black rhinoceroses compared to published wild ranges. Our data show no correlations between insulin, 25(OH)D, TS%, inflammatory markers, or short-chain fatty acids (SFCAs). Serum 25(OH)D exhibited significantly higher levels in summer than in winter. Transferrin saturation was influenced by age, which is consistent with previous studies. The microbiome did not differ significantly among individuals, institutions, sex, or season, unlike the mycobiome, which exhibited significant differences across institutions. The impact of the mycobiome differences on the physiology of the animals could not be determined from this study.

## Introduction

The black rhinoceros (*Diceros bicornis*) is listed as critically endangered by the IUCN (Emslie, 2020). Therefore, several institutions worldwide are working to conserve this species *ex-situ* (Emslie and Brooks, 1999; Pilgrim and Biddle, 2013, 2020). Iron overload disorder (IOD) has been, and continues to be, a significant issue reported among the *ex-situ* population of black rhinoceroses. In the most recent retrospective study on the causes of mortality in 67 ex-situ European black rhinoceroses from 1995 to 2022, IOD was the most common finding (100% prevalence in tested animals over 5 years of age). Iron overload has also been identified in comorbidity (100%) in rhinoceroses diagnosed with dermatitis/dermopathy in general (39% prevalence) and specifically necrolytic skin dermatitis (7% prevalence); pododermatitis (12% prevalence); glomerulopathy/nephritis (34% prevalence); neoplasia (6% prevalence); dental issues (30% prevalence); hemolytic anemia (4% prevalence); and hypophosphatemia (1% prevalence) (Radeke-Auer et al., 2023). Therefore, it cannot be concluded that IOD was the primary cause of death in the European *ex-situ* population. However, its association with many other common pathologies still needs to be considered influential in systemic function.

Although the etiology is not known, the development of IOD in the black rhinoceros could be partly related to differences in zoo diets compared to those in the wild. The black rhinoceros is a browser, feeding on herbs, shrubs, tree leaves, and twigs (Dierenfeld et al., 1995). Browse has low levels of absorbable iron, as it contains relatively high levels of natural Fe-binding substances, such as tannins (Helary et al., 2012). Zoological institutions typically cannot provide a diet consisting solely of browse, and the browse plants that are provided often differ from native ones. Therefore, the nutritional components of zoo diets differ significantly from what is consumed in the wild, resulting in zoo diets potentially containing more absorbable iron (Dierenfeld, 1997; Helary et al., 2009; Candra et al., 2012; Ricketts et al., 2021). Another contributing dietary factor, alongside the high iron content and low levels of natural chelators, may be the reduced amount of antioxidants, as noted by Sullivan et al. (2020). IOD and the previously mentioned conditions have been linked to oxidative stress (Paglia and Tsu, 2012; Pouillevet et al., 2020) and inflammation (Pouillevet et al., 2020).

Next, to the composition of the diet, there is also the gut microbiome. The gut microbiome is an important factor in the health status of that animal (Kogut and Arsenault, 2016). Some researchers consider the microbiome a vital organ not only to digest food but also to develop a fully functional immune system and to act as an endocrine organ through the release of microbially derived metabolites such as short-chain fatty acids (SCFAs) (Wishart, 2019). The extent to which SCFAs are produced depends, amongst other factors, on the composition of the gut microbiome (Flint et al., 2008).

The microbiome of the black rhinoceros has been investigated in a few studies (McKenzie et al., 2017; Gibson et al., 2019; Roth et al., 2019; Antwis et al., 2019; Cersosimo et al., 2022; van der Meijs et al., 2024). McKenzie et al. (2017) and Gibson et al. (2019) demonstrated differences in the microbiome between *ex-situ* and wild black rhinoceroses. Roth et al. (2019) showed differences in the microbiome between IOD-resistant and IOD-susceptible rhinoceros species. Van der Meijs et al. (2024) also showed that zoo-managed black rhinoceroses had a more diverse microbiome per individual compared to white and Indian rhinoceroses. Factors such as season (van der Meijs et al., 2024), age, and sex (Roth et al., 2019) did not appear to have an effect on the microbiome. Although no effect of sex was found in these studies, another research on black rhinoceroses suggested that dysbiosis was linked to the fertility decline of female black rhinoceroses (Antwis et al., 2019).

Metabolomics has been conducted several times in rhinoceroses (Watanabe et al., 2016; Roth et al., 2019; Cersosimo et al., 2022; Corder et al., 2023). In Sumatran rhinoceroses, a species highly susceptible to IOD, the metabolite profiles of *ex-situ* and (semi)wild animals were significantly different (Watanabe et al., 2016). Roth et al. (2019) found that black rhinoceroses had the most variable metabolome compared to white (*Ceratotherium simum*), greater horned (*Rhinoceros unicornis*), and Sumatran rhinoceroses (*Dicerorhinus sumatrensis*) in ex-situ studies; this conclusion was also supported by Cersosimo et al. (2022). Corder et al. (2023) identified a sex difference in propanoate metabolism, and by comparing healthy with diseased black rhinoceroses, they found a difference in the arachidonic acid pathway, hypothesized to contribute to mitochondrial dysfunction by creating oxidative stress.

IOD in black rhinos might resemble the so-called metabolic syndrome seen in human medicine, as both involve insulin resistance, inflammation, and oxidative stress, leading to dysmetabolic iron overload syndrome (Barbalho et al., 2023).

High levels of insulin in *ex-situ* black rhinoceroses have been described by Schook et al. (2015). In horses, another perissodactyl, a positive correlation was found between ferritin and the insulin response (Nelson et al., 2012), indicating a possible link between iron overload disorder and insulin resistance. However, this correlation was most likely caused by one hyperinsulinemic horse (Kellon and Gustafson, 2019). Another study involving mice demonstrated a correlation between diet-induced insulin resistance, increased hepatic iron, and decreased hepcidin expression (Tsuchiya et al., 2013). The inflammatory status of black rhinoceroses has been investigated in several studies (Schook et al., 2015; Pouillevet et al., 2020). Pouillevet et al. (2020) found that levels of α_2_-globulins were significantly higher in *ex-situ* black rhinoceroses compared to white rhinoceroses. Schook et al. (2015) examined tumor necrosis factor α (TNF-α) and serum amyloid A (SAA), concluding that these markers are much higher in *ex-situ* black rhinoceroses than in their wild counterparts. Inflammatory factors such as TNF-α play a key role in regulating insulin/glucose homeostasis by inhibiting glucose uptake in cells, leading to hypoglycemia in rats (Lang et al., 1992; Hotamisligil et al., 1996). Inflammatory factors, including interleukin (IL)-6, also influence hepcidin production in the liver in humans and mice (Binder and Mansbach, 2015). These inflammatory markers have been hypothesized to contribute to the development of IOD in black rhinoceroses (Ganz and Nemeth, 2012). To the authors’ knowledge, hepcidin has not been successfully analyzed in black rhinoceroses.

Another factor that might influence immunity and other physiological processes is the level of vitamin D. Low levels of serum vitamin D have been associated with insulin resistance, obesity, and various metabolic illnesses in humans (Park et al., 2018). Khoo et al. (2011) found that *in vitro*, 1,25(OH)_2_D_3_ downregulates IL-6, tumor necrosis factor-α (TNF-α), IL-17, and interferon-γ (IFN-γ); these authors noted a seasonal effect *in vivo*, as 25(OH)D_3_ levels increased during spring and summer, which suppressed both IL-17 and IFN-γ. In humans, 1,25(OH)_2_D_3_ can also upregulate cytokines such as IL-10 (Nelson et al., 2012). The rhinoceroses in this study were housed at much higher latitudes compared to their natural range and, consequently, encountered less UVB radiation and lower ambient temperatures, both significant for the endogenous production of vitamin D_3_. Two studies showed seasonal variation in *ex situ-housed* black rhinoceroses in the USA (Olds et al., 2018) and Europe (Bruins-van Sonsbeek and Corbee, 2024). Whether this seasonal difference arises from increased dietary vitamin D_2_ or endogenously produced vitamin D_3_ is unknown. Furthermore, it remains uncertain if (black) rhinoceroses can produce vitamin D_3_ endogenously; horses, for instance, cannot (Azarpeykan et al., 2022).

This study aimed to describe the relationships among 25(OH)D, transferrin saturation, inflammatory markers, cytokines, and insulin concentrations in the blood of the animals. It also examined their links to the gut microbiome, SCFAs, medium-chain fatty acids, food components, and seasonal variations, potentially providing further insight into the metabolic disturbances in *ex-situ* black rhinoceroses.

## Materials and methods

Blood samples were collected from black rhinoceroses every 2 months as part of routine health checks conducted at five European zoological institutions. Table 1 provides the details of the rhinoceroses involved in this study. The sample included five male and seven female rhinoceroses. The mean age by sex was 9.4 years for males and 21.3 years for females. The overall age ranged from 0.4 to 39 years, with a mean of 16.3 years at the time of sample collection.

**Table 1:** Details on zoo location, sex, age, and diet of animals.

Institution	Animal number	Sex	Birth date (age in years)	Provided diet
A	1	m	08.11.2001 (19)	Roughage: lucerne silage Browse: willow branches, rose leaves Concentrates: rhinoceros pellet from Vente
	2	f	23.12.2017 (3)	
	3	f	10.12.2011 (9)	
	4	m	08.11.2020 (0.4)	
B	5	m	06.05.2008 (13)	Roughage: lucerne hay Browse: branchesConcentrates: Pellets Granovit rhino
	6	m	20.01.2019 (2)	
C	8	m	03.04.2008 (13)	Roughage: grass, lucerne hay Browse: branches with leaves Concentrates: browser Pellets Mazuri Ele/Vit
	9^†^	f	01.10.1989 (31)	
D	10	f	03.07.1996 (25)	Roughage: lucerne hay Browse: branches with leaves Concentrates: rhinoceros pellet, salvana E-Selen, bread, apple, oats
	11	f	15.09.1981 (39)	
E	12^†^	f	04.04.1991 (30)	Roughage: lucerne hay, grass in the summertime Browse: raspberry leaves, foliage/branches Concentrates: browser Pellets Mazuri Ele/Vit

† Died during the course of the study, rhino number 7 was removed from further statistics since only one sample could be collected prior to death very early after the onset of the study.

From April 2021 to April 2022, these institutions were asked to store samples for this study on a bi-monthly basis and to collect additional fecal samples: 2 × 2 ml heparin plasma, 2 × 500 μl EDTA whole blood, 2 ml serum, and a minimum of 100 g fresh feces stored at −20°C.

The participating animals had individual stables, occasionally sharing the pasture or outdoor enclosure. Feces were collected from both the individual stables and the shared pasture areas, ensuring that samples could be confidently attributed to specific animals. In one case, a mother and her calf were housed together, and their feces could be easily distinguished by the size of the fecal balls. One complete fecal ball was collected and stored in a plastic bag in the freezer for each collection period. The fecal collection was consistently completed within 24 h, and most often within 12 h after defecation.

A total of 63 serum samples were collected from 12 black rhinoceroses across five zoological institutions in Europe; however, only 62 samples from 11 black rhinoceroses were selected for the analyses (individual #7 died from trauma after the first sampling round). Table 2 provides an overview of the samples taken. Two animals died near the end of the study (individual #9 died of end-stage renal failure, with iron stores found in multiple organs; individual #12 had diverticula of the gut leading to severe fecal congestion, with iron stores present in all organs) and contributed to almost all sample periods.

**Table 2:** Samples (serum, heparin plasma and EDTA whole blood) obtained (‘×’ present, ‘-‘lacking) from black rhinoceroses held at five zoological institutions.

Institution	Animal number	April ‘21	June ‘21	August ‘21	October ‘21	December ‘21	February ‘22	April ‘22
A	1	×	×	×	×	×	×	×
	2	×	×	×	×	×	×	×
	3	×	×	×	×	×	×	×
	4	-	×	×	×	×	×	×
B	5	×	-	×	-	-	-	-
	6	×	-	×	-	-	-	-
C	8	×	×	×	×	×	×	×
	9	×	×	×	×	×	×^†^	-
D	10	×	×	×	×	×	×	×
	11	×	×	×	×	×	×	×
E	12	×	×	×	×	-	×^†^	-

† Died during the course of the study.

At the beginning of the study, 37 males and 52 females were housed in 25 zoological institutions participating in the European Endangered Species Programme (EEP) of the European Association of Zoos and Aquaria (EAZA). Thus, this study represented 20% of all EEP institutions and accounted for 13.5% of all *ex-situ* males and 13.5% of all *ex-situ* females within the EEP.

After every one to two sample periods, samples were shipped frozen at −80°C (BioLogistic Services) to the laboratory at the University of Applied Sciences in Leiden, the Netherlands. There, the samples were thawed, split, and refrozen. The frozen samples were then distributed to other laboratories, where they remained frozen at −80°C until analyses were conducted. To minimize environmental contamination, the outer portion of the frozen fecal samples was removed with a saw and discarded. An accurate weight of approximately 100 g of frozen fecal sample was then mixed with 100 ml of Milli-Q water (Merck-Millipore) in a metal blender cup until a smooth paste was achieved for DNA extraction. This sample was divided into two and refrozen. One portion was processed in a single batch for 16S rRNA, ITS, and matK amplicon sequencing, while the other portion was used for short-chain fatty acid analysis.

A brief questionnaire was sent to institutions regarding health status (symptoms of disorders and more) and information concerning the composition of the diet.

### Clinical serological data

#### Inflammatory markers

For ELISA assays of the inflammatory markers tested, we first cloned the genes of the black rhinoceros to create recombinant proteins. The cDNA of TNF-α and SAA was successfully cloned into the expression vector pET16b, after which the proteins were expressed and purified using His-tagged Co-IDA HD Agarose beads (SERVA). The black rhinoceros sequences exhibited homology with their equine mRNA counterparts: TNF-α (90%) and SAA (94%).

The concentrations of the inflammatory markers TNF-α and IL-6 were measured using the MILLIPLEX MAP Equine Cytokine/Chemokine Magnetic Bead Panel (Merck) and Luminex’s xMAP® technology (Luminex/Diasorin, ‘s Hertogenbosch, NL). All incubation and wash steps were performed according to the manufacturer’s instructions. Recombinant TNF-α (from black rhinoceros) and equine IL-6 (provided in the kit) served as standard curves. Data were analyzed using xPONENT software (version 4.2). Recombinant black rhinoceros TNF-α showed good calibration curves in the MILLIPLEX MAP Equine Cytokine/Chemokine Magnetic Bead Panel and was therefore used as a standard in this ELISA. For the multiplex equine assay, IL-6 standards were run in parallel with serially diluted recombinant IL-6 samples in the specified assay buffer, exhibiting visual parallelism with the standard curve without requiring statistical analysis. The lower limits of detection were <0.06 ng/ml for TNF-α and 15 pg./ml for IL-6. Furthermore, insulin was measured using a Mercodia Bovine Insulin ELISA (Mercodia, Winston Salem, NC) along with a bovine insulin standard, as previously described by Schook et al. (2015).

It is important to note that the rhinoceroses in our study were not fasted. Moreover, SAA concentrations were measured using the Tridelta multispecies SAA ELISA (TriDelta Diagnostic Corp., Dublin, Ireland), following the methodology of Schook et al. (2015), with the exception that a recombinant black rhinoceros SAA standard was used. The SAA ELISA assay showed good calibration curves, supporting its application as a standard method in this ELISA. Finally, IFN-γ concentrations were measured using a homemade ELISA, incorporating a recombinant white rhinoceros IFN-γ standard. This assay, published by Morar et al. (2013), was kindly provided by Prof. Dr. V. Rutten (Utrecht University, Section Immunology, Department of Biomolecular Health Sciences, Utrecht, The Netherlands). The lower limit of detection for IFN-γ was 0.4 ng/ml.

All samples from black rhinoceros ex-situ were analyzed together in one run due to the low serum levels; therefore, intra-assay coefficients were not measured. Furthermore, the intra-assay coefficients of variation (%CV) were below 15%, which served as an exclusion criterion for calculations. Samples were quantified using a Five-Parameter Logistic (5PL) curve fitting (my assays desktop version 8.2.28).

Retrospective serum samples from three animals housed at Institution A were selected from their in-house serum bank during the period of clinical illness to test whether inflammatory markers were elevated compared to the period of no clinical illness.

### Serum transferrin saturation

Iron (Fe) and Unsaturated Iron Binding Capacity (UIBC) were analyzed in serum using an automated chemistry analyzer (Beckman Coulter AU-680) at the Universiteits Veterinair Diagnostisch Laboratorium (UVDL) of Utrecht University in the Netherlands. Fe concentrations were measured spectrophotometrically through a redox reaction, utilizing TPTZ as the chromogen. UIBC was determined spectrophotometrically using Nitroso-PSAP as the chromogen. Total Iron Binding Capacity (TIBC) can be calculated by adding the UIBC value to the Fe value (TIBC = UIBC + Fe). Transferrin Saturation (TS%) was calculated from the Fe and TIBC using the formula TS% = (Fe/TIBC) × 100.

### Serum vitamin D

A total of 25(OH)D concentrations were analyzed in serum using the miniVIDAS® (Biomérieux, Marcy-L’Etoile, France) at the laboratory of the Rotterdam Zoo in the Netherlands. The VIDAS 25 OH vitamin D TOTAL assay combines a competitive enzyme immunoassay method with final fluorescent detection (or enzyme-linked fluorescent assay/ELFA), with a detection limit ranging from 20.3 to 315 nmol/L (BioMérieux, 2016).

### Sequence derived data

#### DNA extraction

Feces were stored at −80°C without lipolyzing or DNA shield until analysis. The feces were thawed and blended. DNA for microbial species identification was extracted from a 0.25 g blended fecal sample using the PowerLyser Powersoil DNA extraction kit (Qiagen) after comminuting each fecal sample for 3 × 45 s at 5,500 rpm in a Precellys bead beater (Bertin Instruments). For plant species identification, DNA was extracted using the Quick-DNA™ Fecal/Soil Microbe MiniPrep Kit (ZYMO Research). The DNA concentration was measured fluorometrically with a Qubit 3.0 fluorometer (Thermo Fisher Scientific).

#### PCR amplification

The entire region of the 16S rRNA gene was used for bacterial identification. DNA amplification was conducted by adding 25 ng of fecal DNA to a mixture containing 2 μl of 16S rRNA primers 27F (5′ -AAGRGTTYGATYMTGGCTCAG-3′) and 1497R (5’-ACCTTGTTACGACTT-3′), both at a concentration of 10 μM. Additionally, 25 μl of Phire Green Hot Start II PCR Master Mix was included, and the volume was adjusted to 50 μl with nuclease-free water. Reactions were incubated at 98°C for 30 s, followed by 40 cycles of 5 s at 98°C, 5 s at 51°C, and 25 s at 72°C, concluding with a final extension of 1 min at 72°C.

The identification of fungi was based on ITS2 target sequences. Amplification of fungal species DNA was achieved by adding 25 ng of fecal DNA to a mixture containing 2 μl of primers ITS3 (5’-GCATCGATGAAGAACGCAGC-3′) and ITS4 (5’-TCCTCCGCTTATTGATATGC-3′), both at a concentration of 10 μM; 25 μl of Phire Green Hot Start II PCR Master Mix, with the volume adjusted to 50 μl using nuclease-free water. Reactions were incubated in a thermal cycler (C1000 touch; Biorad) at 98°C for 30 s, followed by 35 cycles of 5 s at 98°C, 5 s at 50°C, and 15 s at 72°C, with a final extension of 1 min at 72°C.

Plant identification was based on the chloroplast-encoded Maturase K (matK) gene. DNA amplification for plant species was performed by mixing 25 ng of DNA with 2 μl of a 10 μM matK-specific forward primer mix and 2 μl of a 10 μM matK-specific reverse primer mix, as outlined by Heckenhauer et al. (2016). Subsequently, 25 μl of Phire Green Hot Start II PCR Master Mix was added, and the volume was adjusted to 50 μl with nuclease-free water. The reactions were incubated in a thermal cycler (Biorad C1000 Thermal Cycler) at 98°C for 30 s, followed by 35 cycles of 5 s at 98°C, 5 s at 46°C, 15 s at 72°C, and a final extension of 5 min at 72°C.

#### Amplicon library preparation and nanopore sequencing

All amplicons were purified using magnetic beads (NucleoMag; Macherey Nagel) with an amplicon-to-bead ratio of 1:1 for ITS2 and matK and 1:0.7 for 16S amplicons, following the manufacturer’s instructions. The concentrations of the purified amplicons were measured with a Qubit 3.0 fluorometer (Thermo Fisher Scientific) and adjusted to 200 fmol in 12.5 μl of nuclease-free water. A sequencing library was prepared for each amplicon type using the Oxford Nanopore SQK-NBD112.96 kit. The final library preparation was loaded onto a Type 10.4 flow cell for each amplicon type and sequenced on a GridION sequencer (Oxford Nanopore) for 72 h.

#### Bioinformatics amplicon sequencing analysis

Amplicon sequences underwent nanopore sequencing, during which adapters and barcodes were removed and demultiplexed from the nanopore reads using Guppy v.6.1.7. Subsequently, amplicons underwent a preprocessing step involving filtration based on amplicon size (1,600 bp for 16S rRNA, 550 bp for ITS, and 800 bp for matK) and required a quality score of at least 12, implemented using Filtlong v 0.2.1 (Wick, 2017). The Kraken2 v2.1.2 (Wood et al., 2019) and Bracken v2.7 software (Lu et al., 2017) were employed to classify the cleaned reads based on an index of archaea, bacteria, fungi, and plants. The archaea index was sourced from ftp://ftp.ncbi.nlm.nih.gov/refseq/TargetedLoci/Archaea/4-3-2023, while the bacteria index was obtained from ftp://ftp.ncbi.nlm.nih.gov/refseq/TargetedLoci/Bacteria/4-3-2023. The fungi index was acquired from ftp://ftp.ncbi.nlm.nih.gov/refseq/TargetedLoci/Fungi/4-3-2023. The matK accessions were retrieved from the BOLD database and batch-blasted against the GenBank accessions obtained by querying “matK” and “plants.” A list of unique matK accessions was compiled from both databases and used as an index for plant species. Species abundances were validated by aligning reads to reference genomes using NanoPipe (Shabardina et al., 2019). The Kraken2 reports, which included taxonomic classifications and abundances, were consolidated, and metadata were appended to create a BIOM file for further processing. Metadata included rhino names, age, sex, TS%, TS-class, sampling period, season, and zoo identifier. Functional analysis was conducted using DRAM (version 1.5.0; Shaffer et al., 2020) to assemble sequences of gut-associated species from the 16S rRNA and ITS data.

#### Short- and medium-chain fatty acid analysis

Frozen fecal samples were sliced into approximately 500 mg portions and extracted with 1,500 μl of methanol containing an internal standard (butanoic acid-d8). After vortexing and centrifugation at 4,400 rpm for 10 min, 1 ml of the supernatant was transferred to a gas chromatography (GC) vial. The sample was injected into a gas chromatograph coupled with mass spectrometry (GC–MS). For this, a Thermo Scientific™ ISQ™ QD Series single quadrupole and a Thermo Scientific TRACE 1300 Series Gas Chromatograph were used. Samples were analyzed on a polar wax column (CP WAX 52CB), applying a 1:50 split and a PTV injection volume of 0.5 μl. In cases where samples were not analyzed immediately, they were stored at −20°C until analysis.

#### Short- and medium-chain data analysis

SCFAs were quantified by integrating the target SCFAs using the instrument supplier software (Chromeleon vs. 7.2.10). The integrated peak areas were then normalized against the peak area of the internal standard and the weight of the sample.

### Statistical analysis

The characteristics of the rhinoceros were described using methods that included standard deviations, frequencies, and percentages. Proportions quantified the fraction of total reads mapped to each taxonomic level, from phylum to species. A Shapiro–Wilk test was conducted to ascertain the normality of the data distribution. The data were not found to be normally distributed. However, a mean was selected to accompany the median for comparison with existing literature. Stacked bar charts depicted the relative abundance of major bacterial, fungal, and plant species for each rhinoceros species, showcasing the top 20 species with distinct colors representing them. Alpha diversity metrics and indices were obtained using the microbiome v 1.26 package. To identify differences in observed, Chao 1, Shannon, and Inverse Simpson diversity based on rhinoceros name, sex, season, and transferrin saturation class, Kruskal–Wallis or Mann–Whitney tests (including Holm false discovery correction) were performed. The transferrin saturation class is defined as being higher or lower than the median value of measured % transferrin saturation (63.8%). Spearman’s rank correlation coefficients were utilized to assess the relationship between SFCAs and inflammatory markers (ggcorrplot 0.1.4.1, Kassambara and Patil, 2023). Principal component analysis (PCA) was conducted on the raw counts after centered log-ratio transformation (Aitchison distance), while principal coordinates analysis (PCoA) was performed on the Bray–Curtis dissimilarity matrix. Before statistical analysis, abundance measures were transformed using CLR to enable analysis by ANCOM-BC (Lin and Peddada, 2020) and PERMANOVA. ANCOM-BC is a statistical framework designed to account for compositional constraints and reduce false discoveries when detecting differences in microbial mean taxa abundances at an ecosystem level based on compositional log ratios. The differential abundance of bacterial (16S), fungal (ITS), and plant species (matK) in association with TS classes was calculated by ANCOM-BC (version 1.4.0) with an adjusted *p*-value of less than 0.05.

Non-parametric multivariate statistical tests were conducted using PERMANOVA. A Kruskal-Wallis or Mann–Whitney U test was conducted using the ggstatsplot package (version 0.12.3, Patil, 2021) to evaluate relationships between TS% and the factors of zoo, sex, age, and season (with summer defined as April to September and winter as October to March), applying Holm correction for multiple testing. Both Kruskal–Wallis and Mann–Whitney U tests (with Holm false discovery rate correction) were conducted to analyze the between-subject data for vitamin D in relation to the factors of the zoo, gender, rhino name, and season (Patil, 2021). A linear mixed-effects model analysis (lme4 1.1–35.3) was used to explore the associations between TS% and variables, including sex, rhino, age, zoological institution, and season. Differences in beta diversity were assessed through permutational multivariate analysis of variance (PERMANOVA), implemented by the Adonis function in vegan v2.6.1 (Oksanen et al., 2019). Differentially abundant OTUs in low TS class versus high TS class rhinoceros species were identified using the ANCOMBC package. Results from the ANCOMBC analyses were presented as a volcano plot. All analyses were conducted using R v4.3.2 (R Core Team, 2019), incorporating the tidyverse (version 2.0.0; Wickham, 2011,Wickham et al., 2019), phyloseq (version 1.28.0; McMurdie and Holmes, 2013), vegan (version 2.5.5; Oksanen et al., 2019), microbiome (version 1.6.0; Lahti et al., 2017), ggstatsplot (version 0.130; Patil, 2021), ANCOMBC (version 2.7; Lin and Peddada, 2020), and tidyplot (version 0.2.1.9000; Engler, 2024).

### Data deposition

The 16S rRNA, ITS2, and matK datasets generated in this study have been deposited in the European Nucleotide Archive (ENA) under project accession number PRJEB80731.

## Results

### Insulin

Since blood sampling took place during routine screening, the animals were not fasted for collection. Insulin levels ranged from 17.0 to 105.7 mIU/l, with a median of 27.0 (Table 3). The medians for females and males were not significantly different (25.9 mIU/l (17.0–55.1) vs. 49.6 mIU/l (23.7–105.7), respectively; *p* = 0.21). Insulin levels differed significantly between rhinoceroses (*p* = 0.01), institutions (*p* = 0.03; *post hoc* analysis revealed a difference between institutions A and B, *p* = 0.02), and age (*p* = 0.02), but not between seasons (*p* = 0.93). *Post hoc* tests did not confirm any significant differences between the individual rhinoceroses or ages.

**Table 3:** Serum Insulin, Inflammation markers, TS%, and vitamin D [25(OH)D].

Serum analyses		Overall mean (±SD)	Mean female (±SD)	Mean male (±SD)	Overall median (range)	Median female (range)	Median male (range)
*n*		*n* = 62, 11^+^	*n* = 36, 6	*n* = 26, 5	*n* = 62, 11	*n* = 36, 6	*n* = 26, 5
Insulin (mIU/l)		44.3 (±12.1)	36.6 (±14.3)	53.6 (±31.5)	27.0 (17–105.7)	25.9 (17–55.1)	49.6 (23.7–105.7)
Inflam SAA (ng/ml) matory markers		2,008.5 (±6,315.1)	3,205.3 (±7,375.2)	572.3 (±767.4)	78.5 (33.6–36, 506.8)	73.8 (40.9–36, 506.8)	200.5 (33.6–1, 891.0)
IFN-γ (ng/ml)		2.83 (±3.7)	3.08 (±3.24)	2.52 (±3.97)	1.24 (0.0–10.83)	1.57 (0.0–7.69)	0.0 (0.0–10.83)
TNF-α (ng/ml)		1.97 *n* = 59, 11 (±3.38)	1.66 *n* = 35, 6 (±3.92)	2.35 *n* = 24, 5 (±4.12)	0.0 *n* = 59, 11 (0.0–9.51)	0.0 *n* = 35, 6 (0.0–8.20)	0.0 *n* = 24, 5 (0.0–9.51)
IL-6 (pg/ml)		12.27 (±40.49)	20.63 (± 47.07)	2.23 (±4.99)	0.0 (0.0–120.50)	0.04 (0.00–120.5)	0.0 (0.0–11.15)
TS%^*^		65.1 *n* = 60, 11 (±23.63)	74.0 (±24.22)	54.5 *n* = 24, 5 (±12.73)	59.6 *n* = 60, 11 (32.8–99.2)	83.9 (32.8–99.1)	53.2 *n* = 24, 5 (35.9–99.2)

25(OH)D (nmol/l)		233.9 *n* = 58, 11 (±270.6)	316.4 *n* = 34, 6 (±303.5)	135.1 *n* = 23, 5 (±48.9)	159.6 *n* = 57, 11 (23.0–1,710.0)	155.7 *n* = 34, 11 (23.0–1,710.0)	159.6 *n* = 23, 5 (39.5–292.3)

^*^TS% = (Fe/TIBC) × 100. ^**^All adults, juveniles 60 ± 21 (*n* = 26–30). ^***^25(OH)D3. ^****^Boma in Africa.

### Inflammatory markers

The median SAA levels were 78.5 ng/ml, exhibiting a broad range from 33.6 to 36,506.8 ng/ml. IFN-γ presented a median level of 1.57 ng/ml with a range of 0.0 to 7.69 ng/ml. TNF-α had a median level of 0.0 and a range from 0.0 to 9.51 ng/ml. IL-6 showed a median level of 0.0 pg./ml with a range from 0.0 to 120.50 pg./ml (Table 3). All inflammatory markers significantly differed among the individual rhinoceroses and by age (*p* < 0.05), but only TNF-α and IL-6 showed differences based on zoological institution (SAA *p* = 0.06; IFN-γ *p* = 0.25; TNF-α and IL-6 *p* < 0.001). Interleukin-6 was the sole inflammatory marker that significantly differed between the sexes (*p* < 0.01) (Table 3). No significant differences between seasons were detected for any of the inflammatory markers.

### Serum transferrin saturation

**Figure 1:**
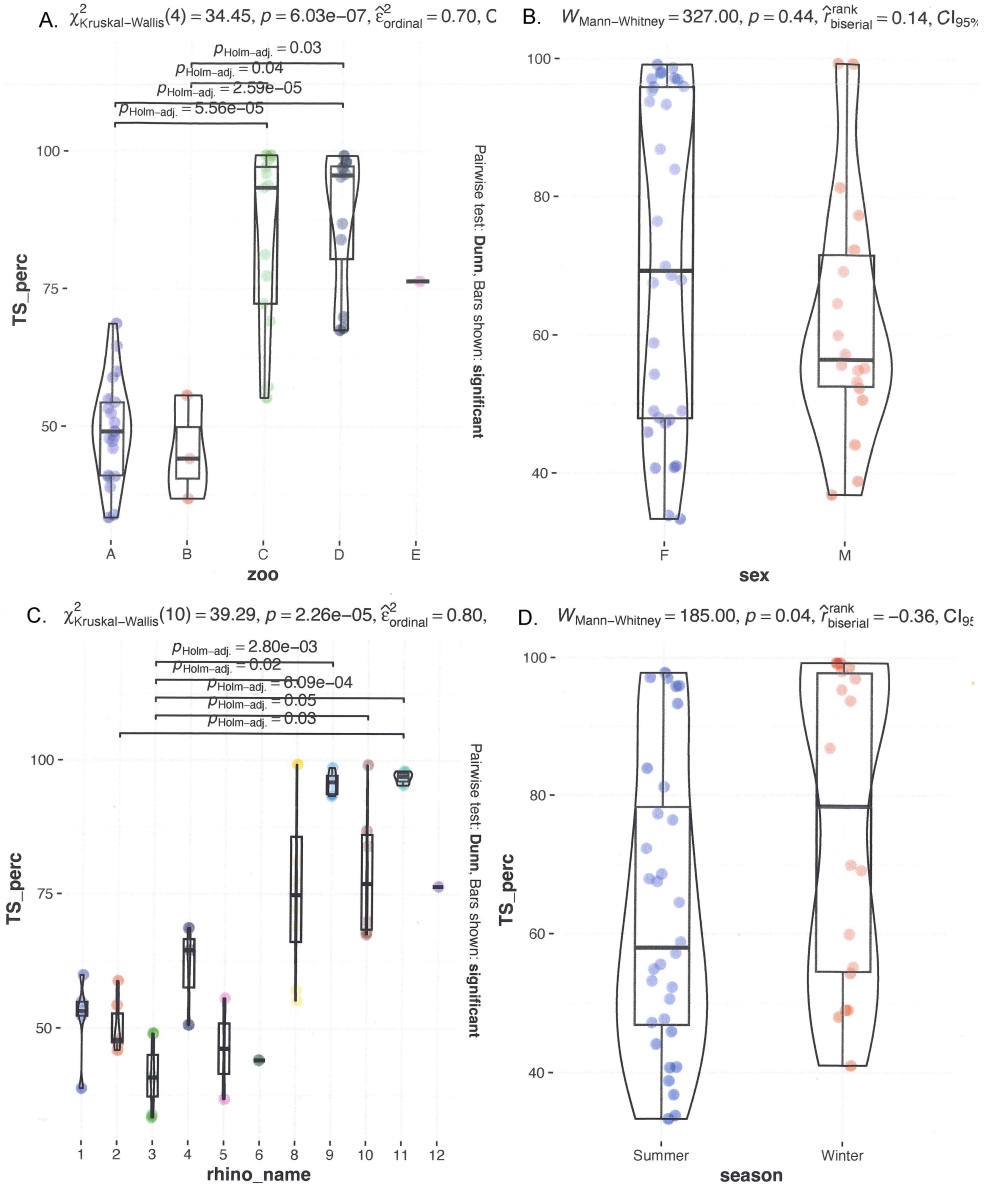
Comparison of serum TS% in different seasons and individual rhinoceroses, and grouped by zoo and sex. On top, left **(A)** grouped by zoo; **(B)** grouped by sex. On the bottom left **(C)** grouped by rhino_name and right **(D)** seasonal distribution.

The median TS% was 59.6%, with a range of 32.8 to 99.2%. Figure 1 shows multiple graphs depicting the distribution of serum TS% grouped by individual rhinoceroses, institution/zoo, sex, and age. TS% was significantly different among individuals and when animals were grouped by zoological institution, significant differences were observed from A to C, D, and E (all *p* < 0.01) and from B to C and D (*p* = 0.2 and < 0.01, respectively) and by age (3–9 years differed significantly from 25 to 39 years, *p* ≤ 0.03; Figure 1). A trend analysis between age and median TS% revealed a correlation coefficient of 0.61, indicating a moderately positive relationship. However, when age and TS% are averaged per institution, a distinction arises between low (< 62.8%) and high (> 62.8%) TS% classes, separating institutions A and B from institutions C, D, and E. The two classes exhibited a significant difference from one another. Examining individual rhinoceroses, significant differences were found among rhinoceroses (no. 2 vs. no. 9 (*p* = 0.02) and 11 (*p* < 0.001); no. 3 vs. no. 8, 9, 10, 11, and 12 (*p* = 0.04, < 0.001, 0.02, < 0.001, and 0.02, respectively)). The median for females was 83.9% (32.8–99.1%) compared to 53.2% (35.9–99.2%) for males. No significant differences were detected based on sex (*p* = 0.07) or season (*p* = 0.16).

### Serum vitamin D

**Figure 2:**
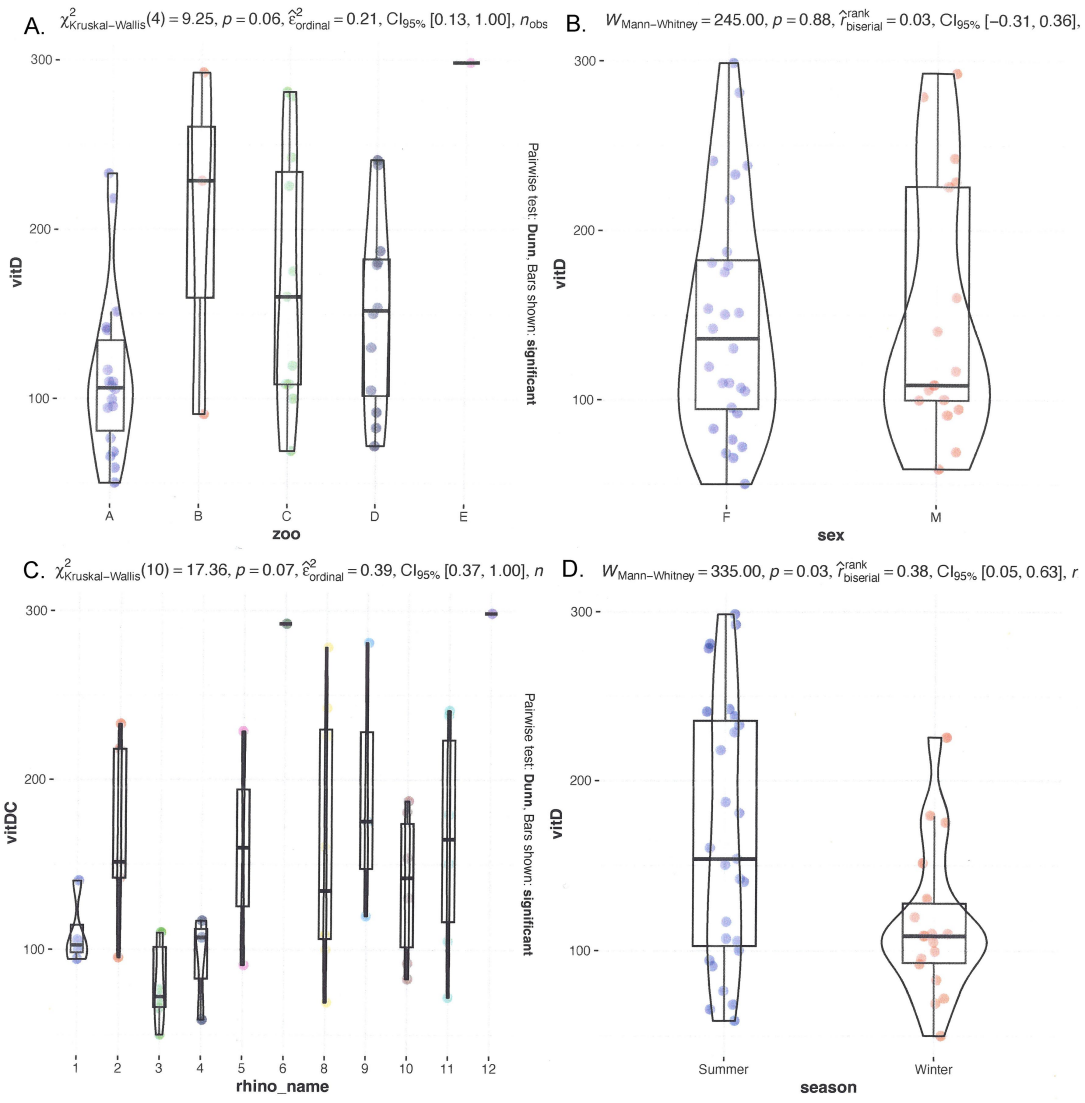
Comparison of serum 25(OH)D in different seasons and individual rhinoceroses, and grouped by zoo and sex. On top, left **(A)** grouped by zoo; **(B)** grouped by sex. On the bottom left **(C)** grouped by rhino_name and right **(D)** seasonal distribution.

The median 25(OH)D levels were 159.6 nmol/L, with a range from 23.0 to 1,710.0 nmol/L. Figure 2 shows multiple graphs showing the distribution of serum 25(OH)D grouped by individual rhinoceroses, institutions/zoos, sex, and season. Significant differences were observed when comparing the median 25(OH)D levels of individual rhinoceroses and zoological institutions (Figure 2). Rhinoceros no. 9 and no. 12 exhibited significantly higher median values compared to most other rhinoceroses (*p* = 0.03). Females displayed a median of 155.7 nmol/L (range 23.0–1,710 nmol/L), while males had a median of 159.6 nmol/L (range 39.5–292.3 nmol/L), which was not significantly different (*p* = 0.26). 25-Hydroxy-vitamin D levels in winter (95.3 nmol/L, range 39.5–294.0 nmol/L) were significantly lower than those in summer (170.6 nmol/L, range 23.0–1,710.0 nmol/L) (*p* = 0.04).

### Sequence DATA

#### Microbiome

**Figure 3:**
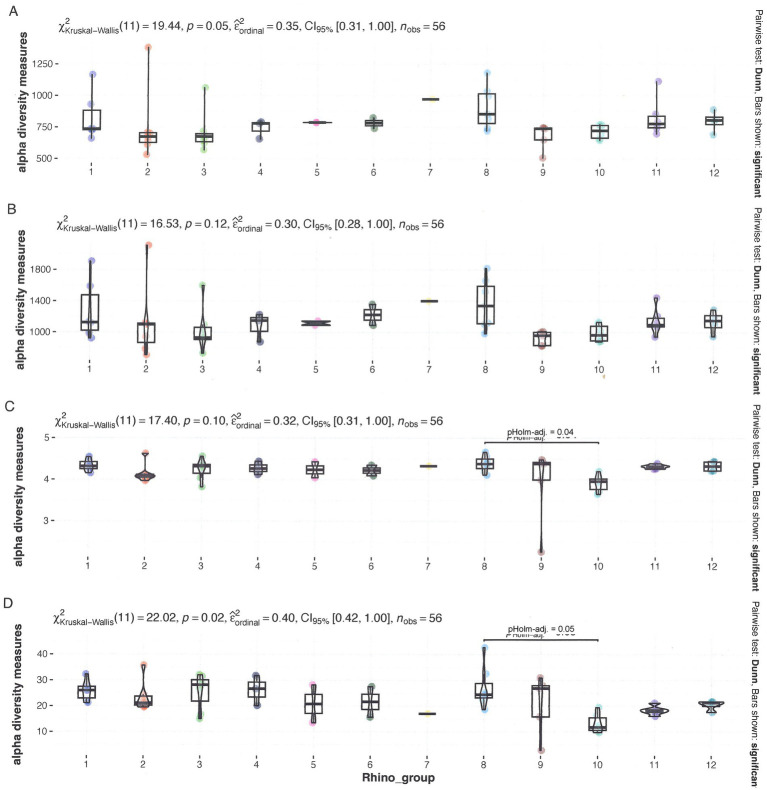
Microbial α-diversity according to grouped rhino_name data. **(A)** Observed diversity **(B)**, Chao1 diversity **(C)**, Shannon diversity, and **(D)** Inverse Simpson. All data have been tested for significant differences by a Kruskal-Wallis test at *p* = 0.05.

The composition of the fecal bacterial population was determined through nanopore sequencing of the full 16S rRNA gene. Supplementary Figure 1 provides the microbiome of each rhinoceros at each sampling period. Three of the most prevalent species were identified during specific time periods associated with bouts of infections. These include *Acinetobacter sichuanensis*, *Kurthia massiliensis*, and *Sharpea azabuensis*. The remaining 17 species are associated with the gut. Two species, *Sporobacter termitides* and *Pipillobacter cinnamivorens*, have been associated with cellulose and tannin degradation, respectively. Figure 3 and the Supplementary Figures 4–7 present the analysis of the relationship between α-diversity and rhinoceros name, sex, age, and TS class. The Inverse Simpson index revealed significant variations for all factors, while sex showed significant differences across all indices.

**Figure 4:**
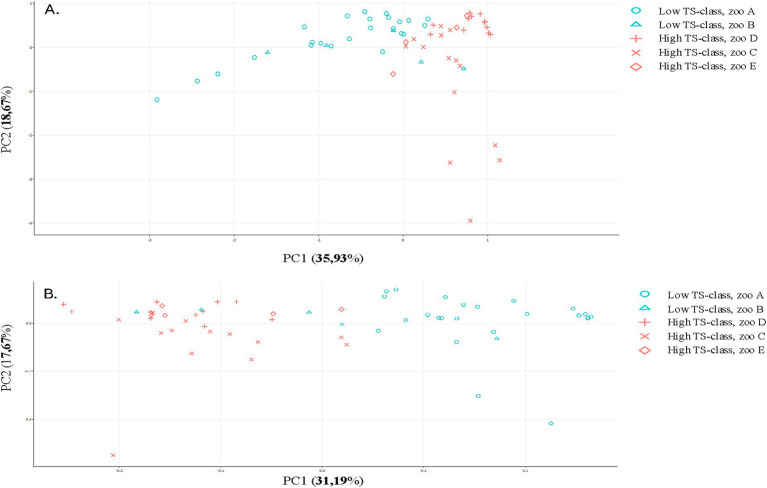
Principal components of rhino fecal samples analyzed by 16S for selected distance matrices. **(A)** Aitchison distance and **(B)** Bray-Curtis dissimilarity.

**Figure 5:**
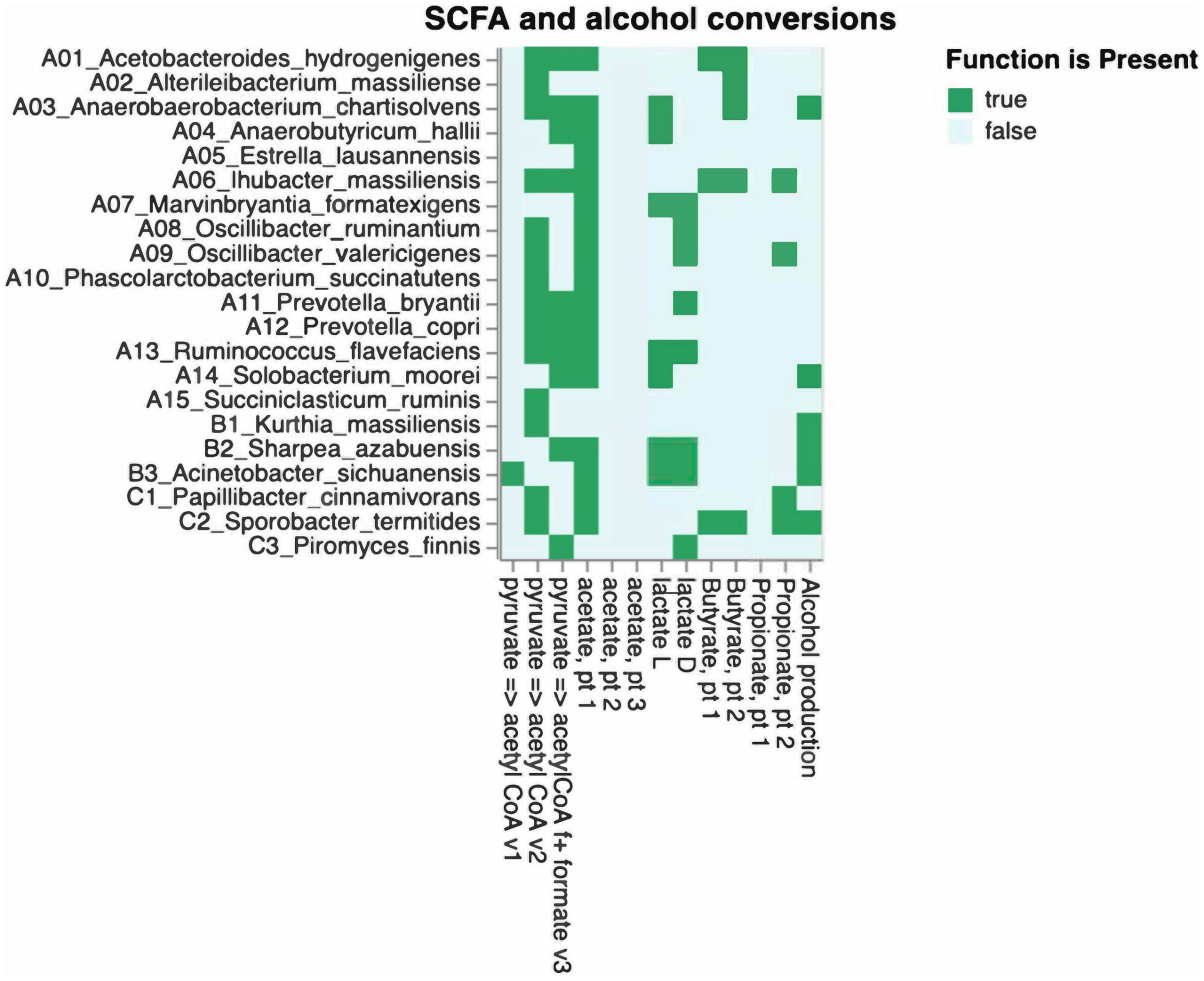
Relation between gut-related bacteria, fungi, and predicted SCFA conversions.

The β-diversity exhibited a division of groups, with the low TS class and zoos A and B on one side and the high TS class along with zoos C, D, and E on the other side, as illustrated in an Aitchison ordination. However, when a Bray–Curtis dissimilarity ordination was applied, this distinction became less pronounced, as some samples from Zoo B intermixed with the high TS class samples (see Figure 3). The ANCOM-BC method was utilized to evaluate the differential significance in composition. A contrast was formed by splitting the TS% data into two sets: one below and one above the median value of 62.8%. While microbiome diversity did not correlate with TS%, certain bacteria appear to be associated with high (*Prevotella, Sharpea, Clostridium, Mogibacterium,* and *Christensella*) or low TS% (*Anaerobutyricum, Succiniclastium, Acinetobacter, Solobacterium,* and *Kurthia*; see Figure 4, *p* < 0.05). The analysis indicated that some bacteria could produce specific SFCAs while others could not (see Figure 5). Data on energy metabolism, sulfur, nitrogen, carbon metabolism, and methanogenesis are demonstrated in Supplementary Figure 19.

#### Mycobiome

The presence of fungi in the fecal samples of the black rhinoceros was determined by nanopore sequencing of the internal transcribed spacer 2 (ITS2) amplicon. Supplementary Figure 2 provides an overview of the mycobiome for each individual rhinoceros during each sample period. The most prevalent fungi can be categorized into two groups: those associated with digestive processes in the gastrointestinal tract and those that function as plant pathogens. The former group comprises *Piromyces finnis, Feramyces austinii, Oontomyces anksri, Pecoramyces ruminantium, Buwchfawromyces eastonii,* and *Anaeromyces mucronatus*. These organisms have been isolated from the feces and rumen of both undomesticated herbivores and captive wild animals, including sheep, goats, cows, buffalo, camels, elephants, and gorillas. All these fungal species belong to the *Neocallimastigaceae* family, which is an order of anaerobic gut fungi primarily found in the digestive systems of herbivorous mammals (Gruninger et al., 2014). These organisms play a pivotal role in the breakdown of complex plant materials, particularly cellulose, through fermentation processes.

Fermentation by *Neocallimastigaceae* produces various metabolites, including fatty acids, gases, and other compounds, contributing to the overall energy metabolism of the host. The α-diversity exhibited significant disparities in richness based on rhinoceros, age, and TS class; however, these disparities were not observed when evenness was considered (see Supplementary Figures 8–11). The β-diversity exhibited a division between groups of low TS class and zoos A and B on one side and high TS class and zoos C, D, and E on the other, as illustrated in an Aitchison ordination. This division becomes less pronounced when utilizing a Bray–Curtis dissimilarity ordination. ANCOM-BC was used to measure the differential significance in the composition of the contrast. A contrast was created by dividing the TS% data into a set below and above the median value of 62.8%. While microbiome diversity was not correlated with TS%, the presence of certain fungi was associated with high TS%, e.g., *Feromyces, Buwfawromyces,* and *Oontomyces* (Supplementary Figure 21, *p* < 0.05).

#### Plant residues

Chloroplast (cp) DNA markers, such as matK, rbcL, and trnL (UAA), have been proposed as standard DNA barcoding markers and have been successfully used in various plant groups to address species identification, phylogenetic connections, and sample origin. In rhinoceros diet studies, rbcL (Kgopa, 2009) and trnL (Harvey, 2021; Harvey Sky et al., 2024) have been previously employed. The presence of plant species in fecal samples was identified through nanopore sequencing of matK amplicons. The matK marker was selected for its extensive coverage of plant species and its ability to generate long amplicons, thereby enhancing specificity. The marker’s specificity was validated by analyzing the diet of Zoo A (see Supplementary Figure 23) and checking whether the ingredients of the recipes from other zoos were detected. In addition to these observations, we noted the presence of plants cultivated in the immediate vicinity of the subjects. Supplementary Figure 3 presents an overview of the top 20 plant species. The α-diversity exhibited significant disparities in the richness of rhinoceros and age; however, these disparities were not observed when evenness was included (see Supplementary Figures 12–15). The β-diversity exhibited a division of groups between the low TS class and Zoos A and B on one side and the high TS class and Zoos C, D, and E on the other side, as depicted in an Aitchison ordination. This division becomes less pronounced when utilizing a Bray–Curtis dissimilarity ordination, as the low TS class becomes partly mixed with high TS class samples from Zoo D. A remarkable finding is that Zoo D includes *Salix* species in the first three samples but not in the last three samples (Supplementary Figure 3; Zoo D). The differential abundance of plant species for the TS class shows an association of *Rosa spinossima, Salix trinadra, Alopecurus aequalis, Potentilla anserina,* and *Poa trivialis* with the low TS class, and *Clematis vitalba, Acer platanoides, Elaeagnus umbellata, Corylus avellana, Cornus sanguinea, Prunus spinosa, Daucus carota,* and *Rubus idaeus* with the high TS class. *Medicago sativa* is significantly abundant but not above a twofold range. Of all plant species, only the effect of rose leaves (*Rosa*) was significant for Institution A (Figure 4).

#### Short- and medium-chain fatty acids

The concentrations of SFCAs and medium-chain fatty acids found in the feces of rhinoceroses were very comparable across the various institutions (see Supplementary Figure 22). Overall, SCFAs constituted 91%, compared to 9% for medium-chain fatty acids. Acetic acid was the most prevalent, contributing approximately 50% to the total of SCFAs and medium-chain fatty acids. Butyric acid accounted for 24%, while propionic acid made up 16% of the analyzed fatty acids. Pentanoic acid was the most common medium-chain fatty acid, comprising 7% of the analyzed fatty acids.

#### Correlations and associations

**Figure 6:**
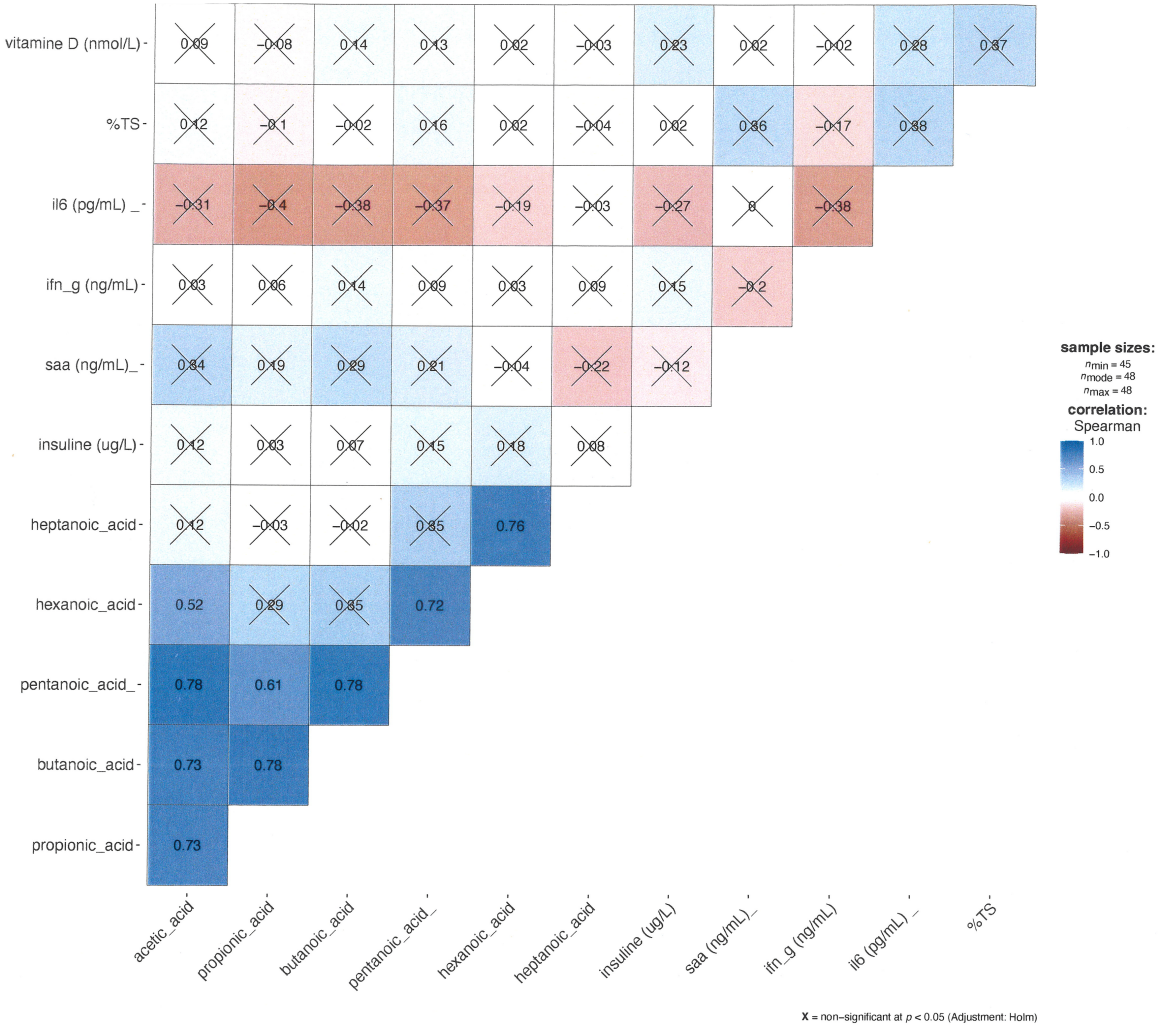
The correlation matrix between the short-chained fatty acids and serum analyses: darker blue indicates a stronger Spearman correlation, and darker red is closer to -1 (stronger negative Spearman correlation). The alpha-level was set to *p* = 0.05. I have marked non-significant correlations with ‘X’.

Figure 6 displays a Spearman correlation matrix of all serological markers and short- and medium-chain fatty acids. The analysis reveals correlations are observed exclusively among short-chain fatty acids, while no significant correlation is observed between inflammatory markers or between short-chain fatty acids and inflammatory markers.

#### Linear models

A linear model for transferrin saturation (TS%) was developed by considering the factors of age, sex, zoo, rhino_name, and season. Although the TS% distribution is not normal, a linear model can still be constructed if the residuals of the fitted model are normally distributed. A Q-Q plot was created to identify outliers, leading to the removal of three outliers from the TS% data. Initially, an additive model was developed, incorporating log transformations, Box-Cox transformations, and generalized linear model (glm) transformations. In the second phase, interaction terms were also included. The optimal model was chosen based on the lowest Akaike Information Criterion (AIC) and Bayesian Information Criterion (BIC) values. The best fit (adjusted r^2^ = 0.78) was identified as %TS ~ age*rhino_name + zoo + sex + season. Subsequently, the distribution of the residuals was analyzed using a Shapiro test, which confirmed that the residuals are normally distributed. A Breusch-Pagan test indicated that the data is homoscedastic.

## Discussion

This study aimed to describe the relationships between 25(OH)D, transferrin saturation, inflammatory markers/cytokines, and insulin concentrations in the blood of the animal, as well as their connections to the gut microbiome, SCFAs, medium-chain fatty acids, food components, and season, to potentially gain further insight into the metabolic disturbances in *ex-situ* black rhinoceroses. It was found that the overall levels of inflammatory markers and insulin were higher compared to values reported in the literature for black rhinoceroses living in the wild, corroborating previous studies conducted on *ex-situ* rhinoceroses (Table 3).

**Figure 7:**
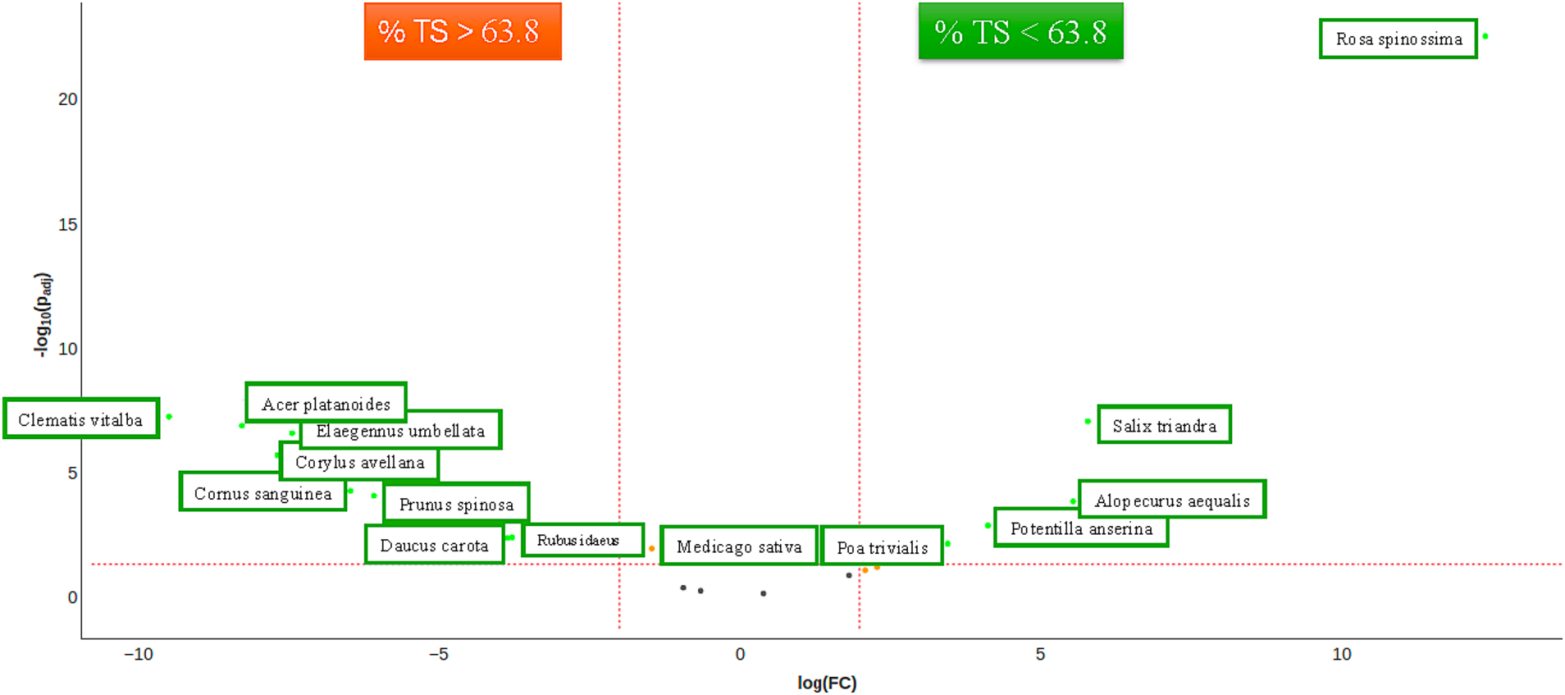
Significant abundance of plant species (matK, FDR < 0.05) between rhinoceroses with low TS% (<63.8%). The threshold of 63.8% was taken from the median TS% of the population under study. The significant abundances are visualized as a Volcanoplot. The orange dot represents significant abundance, and the green dot represents both significant and fold changed >2 of plant species concerning TS class.

Diagnosing IOD ante mortem is very difficult. Transferrin saturation is considered one of the most sensitive indicators of Fe overload in mammals ante mortem; a TS% value of 20–50% is considered normal for the majority of mammals (Piperno, 1998; Sullivan et al., 2020; Lowenstine and Munson, 1999; Bohn, 2013; Lowenstine and Stasiak, 2015). When it exceeds 50%, ferritin levels typically increase, as does the Fe load (Piperno, 1998; Sullivan et al., 2020). Ferritin has been analyzed in rhinoceroses across various studies and has been found to be an unreliable parameter for diagnosing IOD ante mortem (Roth et al., 2017; Wojtusik and Roth, 2018). Therefore, we chose not to analyze ferritin levels in this study. The median TS% of the rhinoceroses in this study was 62.8% (range 32.8–99.2%), indicating that a significant portion of the animals had a high iron load (Figure 7). More recent studies have explored other markers to determine IOD ante mortem, such as serum labile plasma iron (Roth et al., 2022) and serum microRNAs (Wojtusik et al., 2021), with the latter identifying several potentially promising new biomarkers. These biomarkers were published after the laboratory work for the present study had concluded; therefore, we could not assess these specific markers.

The present study demonstrated that when rhinoceroses were grouped by age, there were significantly higher %TS in older animals (those greater than 33 years old) compared to those under 10 years old. A previous study on black rhinoceroses found that TS% increased with age (Smith et al., 1995). In contrast, a recent study found no increase in ferritin with age, but it did not assess TS% (Wojtusik and Roth, 2018). The higher values observed at a very young age (0–2 years) might indicate that these animals were born with adequate Fe, a point also raised by Kock et al. (1992). Our linear model indicated that the institution may be a confounding factor, involving multiple aspects such as enclosure size (potentially related to activity), group composition, diet, and other husbandry factors.

When grouping the animals per zoo/institution, three (C, D, and E) out of the five institutions had significantly higher TS% levels. These three institutions also housed the oldest animals in the present study (Table 1; C 13–31 years, D 25–39 years, E 30 years; compared with A 0.4–19 years and B 2–13 years). Whether age or other zoological aspects of the institution are of more important influence cannot be decided based on the current data.

Due to damage from free Fe radicals, IOD animals are more susceptible to inflammation and diseases, including metabolic syndrome (Sullivan et al., 2020). Pouillevet et al. (2020) demonstrated high levels of inflammatory markers in *ex-situ* European black rhinoceroses. They suggested that either the high inflammatory status might cause IOD or the elevated inflammatory status is a result of IOD. The present study was unable to demonstrate a correlation between insulin and the inflammatory markers (SAA, IL-6, TNF-α, and IFN-γ), TS% and 25(OH)D. This may be due to a significant portion of the studied rhinoceroses exhibiting very high TS% values. This also resembles the findings from the necropsy evaluations by Radeke-Auer et al. (2023).

The SAA measured in the present study was generally higher than that of both *ex-situ* and free-ranging animals reported by Schook et al. (2015). In a study involving 11 *ex-situ* black rhinoceroses, Rispoli et al. (2025) found that clinically healthy rhinos had low or undetectable SAA levels (< 1,000 ng/ml), subclinically ill rhinoceroses exhibited concentrations between 1,000 and 7,000 ng/ml, and clinically abnormal rhinoceroses presented concentrations exceeding 7,000 ng/ml. They employed the same detection method but utilized different laboratories. In our study, one of the rhinoceroses (number 12) had concentrations ranging from 948 to 36,507 ng/ml throughout the year, and three animals showed single analyses exceeding 1,000 ng/ml but below 7,000 ng/ml (rhino number 4: 2,660 ng/ml; animal number 6: 3,735 ng/ml; rhino number 9: 1,065 ng/ml).

The TNF-α levels follow the same trend as the SAA levels. Differences in the levels between the *ex-situ* populations might be explained by the use of different laboratories (the same techniques/kits have been used for all inflammatory markers and insulin), differences in animal husbandry, or individual variation.

Interleukin 6 stimulates hepcidin expression, leading to decreased iron absorption in humans (Binder and Mansbach, 2015). However, this was not supported by this study, as IL-6 showed no correlation with TS%. Future studies investigating the effects of inflammation and Fe metabolism should focus on analyzing hepcidin rather than TS%.

The insulin level of the rhinoceroses reported here was much lower than the *ex-situ* animals by Schook et al. (2015) but still considerably higher than the free-ranging animals despite using the same assay (but a different laboratory). Differences in insulin found by Schook et al. between *in-* and *ex-situ* populations might be due to the development of insulin resistance in captivity. This is also supported by Wojtusik et al. (2021), who have discovered that the expression of miR-143 was higher in rhinoceroses with IOD compared to rhinoceroses that, most likely, did not suffer from IOD. In mice, this specific microRNA has been associated with disturbances in glucose metabolism and alterations in insulin sensitivity (Jordan et al., 2011), and it was also hypothesized to be involved in the development of diabetes type II in humans (reviewed in Li et al., 2018). It should be noted that the animals in the current study were not fasted (which likely altered glucose and insulin levels), and also glucose was not analyzed. Future research into rhinoceros insulin should consider fasting and measuring several blood glucose indicators.

Higher levels of 25(OH)D in summer compared to winter, as reported by Olds et al. (2018) and observed here, suggest that some black rhinoceroses are unable to produce 25(OH)D_3_ endogenously during winter in the Northern Hemisphere. This finding is further supported by a comparison with the 25(OH)D levels found in wild black rhinoceroses in Clauss et al. (2005), where the median of the winter values in this study was considerably lower, while during the summer months, they exceeded the mean levels observed in wild animals.

The comparison of levels of 25(OH)D must be made with caution, as we employed an enzyme-linked fluorescent assay, while other studies used different techniques: Clauss et al. (2005) used a vitamin D binding protein assay, and Olds et al. (2018) employed a radioimmunoassay. Cavalier et al. (2014) compared 25(OH)D analyses across various immunoassays and concluded that these methods are not comparable, especially when levels of vitamin D binding protein are altered, such as during pregnancy, hemodialysis, and osteoporosis in humans. Notably, we did not differentiate between the D_2_ and D_3_ forms, leaving it unclear whether the seasonal effects are indicative of UVB action on plant material (D_2_ formation) or on animals (vitamin D_3_ formation).

Another possible explanation for the seasonal difference is that the roughage was offered outside during the summer months, which likely resulted in increased UVB exposure, thereby raising the level of vitamin D_2_ compared to roughage given in the winter (Opsomer et al., 2025). This accounts for the seasonally fluctuating vitamin D serum levels in horses despite their apparent inability to produce endogenous vitamin D_3_ (Azarpeykan et al., 2016, 2022). This finding is also supported by recent research involving another hindgut fermenter, the rabbit. Rabbits fed UVB-irradiated roughage displayed significantly higher 25 (OH) D levels compared to those fed non-irradiated roughage (Mäkitaipale et al., 2024). Furthermore, a recent study indicated that the microorganisms in the digestive tracts of mice, sheep, and cows seem capable of producing vitamin D_2_ (Chaves et al., 2024), though no additional details were provided regarding the microbiome in this particular study. Given that microbiome synthesis of vitamin D is independent of UV irradiation, it may still contribute to vitamin D levels when UV exposure is insufficient, making it a worthy area for further investigation. Although no correlation was found between 25(OH)D and TS% or with the inflammatory markers, a suggestion for future research on vitamin D would be to distinguish between 25(OH)D_2_ and D_3_. This differentiation would shed light on whether (black) rhinoceroses can produce endogenous vitamin D_3_ and, if they can, whether this production is adequate for maintaining year-round vitamin D levels.

**Figure 8:**
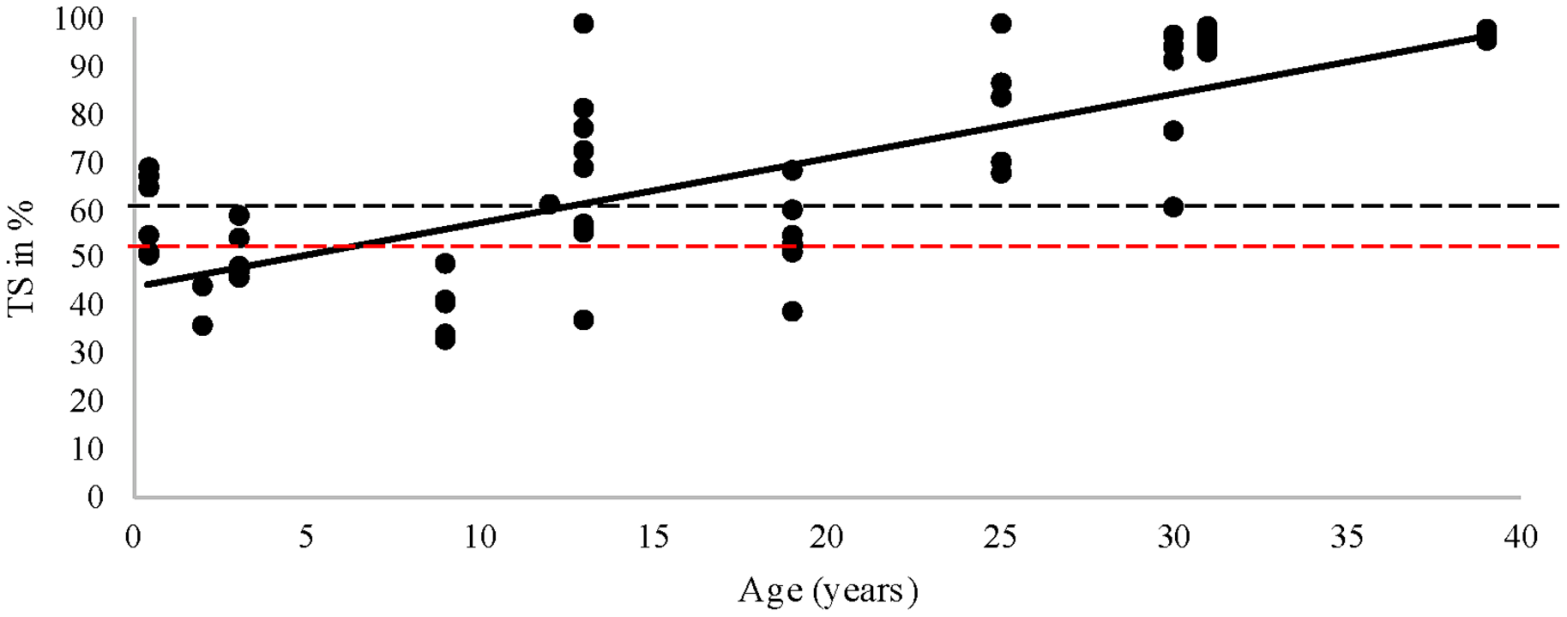
Transferrin saturation (TS) vs. age in years. The black dots illustrate every unique sample, the black line shows the trend, the red striped line is a TS of 50%, the black striped line is a TS level of ~60%, and the median of the rhinoceroses in the current study.

Gibson et al. (2019) identified the top three phyla of the microbiome in both *ex-situ* and wild black rhinoceroses as *Firmicutes* (51.5% in the wild vs. 48% in *ex-situ*), followed by *Bacteroidetes* (17.6% in the wild vs. 42.4% in *ex-situ*) and *Proteobacteria* (23.8% in the wild). The data reported in this study, with the top three bacterial phyla being *Firmicutes* (80.1%), *Bacteroidetes* (14.0%), and *Proteobacteria* (2.28%) (Figure 8), align closely with the findings from the *ex-situ* rhinoceroses; however, our values for Firmicutes are significantly higher, while those for *Proteobacteria* and Bacteroidetes are notably lower. Additionally, Roth et al. (2019) found that *Firmicutes* (51–66.3%) and *Bacteroidetes* (23.4–39.8%) were the most prominent phyla in the rhinoceros microbiome. In their study, the IOD-susceptible species (black and Sumatran rhinoceroses) demonstrated a much higher mean log ratio of *Firmicutes* to *Bacteroidetes* compared to the non-susceptible species (white and greater one-horned rhinoceroses). Antwis et al. (2019) also confirmed that Firmicutes were among the two most abundant phyla, followed by *Bacteroidetes*. This finding was echoed by van der Meijs et al. (2024), which reported that *Firmicutes* (46.8 ± 7.37%) were the most abundant, followed by *Bacteroidetes* (26.9 ± 5.14%) across all rhinoceros species (black, white, and greater one-horned). In contrast to the results of the present and other studies, Cersosimo et al. (2022) reported a higher level of *Bacteroidetes* compared to *Firmicutes* (49.2% vs. 26.2%) in *ex-situ* black rhinoceroses. They concluded that this difference between wild and *ex-situ* black rhinoceroses could be attributed to variations in diet composition.

Van der Meijs et al. (2024) could not demonstrate a difference in the microbiome of *ex-situ* rhinoceroses between summer and winter; however, they did reveal a relationship with their feeding preferences (browsers were clustered together, whereas grazers were found in the white rhinoceros, and intermediates were found in the greater one-horned rhinoceros). Additionally, individuals from different zoological institutions were more clustered together, which may indicate a connection to varying diets. Hoffman et al. (2013) investigated the associations between diet and microbiome in humans and reported that changes in the microbiome correlated more strongly with long-term dietary changes. In the present study, a significant difference was found in microbiome diversity based on sex (not institution). This supports the findings of Antwis et al. (2019), who also identified a difference in the microbiome related to fertility status, as some females in this study were reproductively active. Moreover, genera such as *Sporobacter* and *Pipillobacter,* recognized for their cellulose and tannin-degrading abilities, were identified among the top 20 species. At the species level, 17 species were detected in all samples, although their ratios varied per sample. The remaining three species (*Kurthia massiliensis*, *Acinetobacter sichuanensis*, and *Sharpea azabuensis*) were only observed in samples with high SAA levels, indicating they may be considered abnormal rather than part of the normal intestinal flora.

In our study, we found acetate to be the most prevalent short-chain fatty acid (SCFA), accounting for 48 to 56%, followed by butyrate (21 to 25%) and propionate (15 to 17%). This contrasts with the findings of Cersosimo et al. (2022), who reported that propionate was more abundant than butyrate after acetate, while acetate remained the most prevalent SCFA in their study. This discrepancy may be attributed to differences in the Bacteroidetes-to-Firmicutes ratio observed across both studies. *Bacteroidetes* primarily produce propionate, while *Firmicutes* generate butyrate alongside propionate (Houtman et al., 2022). SCFAs, particularly butyrate and propionate, play an important role in gut homeostasis and overall health. Notably, butyrate has been shown to enhance glucagon and insulin expression in the pancreas (reviewed in Wishart, 2019).

Metabolomics has been applied in the study of Sumatran rhinoceros (Watanabe et al., 2016). These authors compared the metabolite profiles of three captive Sumatran rhinos with those of four wild or semi-wild Sumatran rhinos that died at an Asian sanctuary. They found a significantly different metabolite profile. For the *ex-situ* animals, they also compared samples from diseased and non-diseased animals, identifying 18 altered metabolites, including amino acids and fatty acids (Watanabe et al., 2016). Corder et al. (2023) described differences in the metabolomic profiles of ex-situ black rhinos with and without clinical inflammatory phenotypes, identifying perturbations in the pentose phosphate, arachidonic acid, and bile acid biosynthesis pathways that lead to altered redox signaling and mitochondrial malfunction. Bile acids are primarily associated with nutrient absorption; however, they also serve additional functions in immunomodulation and the regulation of glucose, lipid, and energy metabolism (Xie et al., 2021). Our study did not reveal significant differences in SCFAs over a one-year period in the same animals nor between different institutions. This may be due to the limited number of individuals and their overall high levels of inflammatory markers, insulin, and TS%, which could indicate that all individuals already had metabolic disturbances despite their differences in age, sex, and diet. Another explanation might be that the present study included a targeted list of metabolites. Using an untargeted (global) metabolomic profiling approach may yield significant associations with other metabolites not covered by short- or medium-chained fatty acid analysis.

In rhinoceroses, there has been only one study that included the mycobiome in a shotgun study by Gibson et al. (2019). However, we could not draw any conclusions from this data concerning our own findings. Although the microbiome did not differ, the mycobiome varied significantly across different institutions (Figure 3). The most prevalent gut-associated fungi belong to the *Neocallimastigaceae* family, known for its cellulose degradation function. In other studies, the mycobiome has been found to play a role in the host’s inflammatory response (McKenzie et al., 1990; Li et al., 2014). Institutions A, B, and E showed mycobiomes that were more comparable to each other than to institutions C and D. Institutions A, B, and E also exhibited the highest levels of SAA, TNF-α, and IL-6 (not shown), as well as 25(OH)D (Figure 2). Whether this is related to the mycobiome cannot be concluded from this study.

MatK successfully identified all plant-based materials in the diet, revealing a potential relationship between different types of plants and high or low TS%, as in the results (Figures 6, 9). Among the plant species analyzed, only the effect of *Rosa* was significant at institution A (see Supplementary Table 1, Figure 6). However, *Rosa* was also detected at Institution E, where the species fed was rose (*Rosa*) vs. raspberry (*Rubus*) at Institution E.

Plant components such as tannins, which act as a natural chelator, were not analyzed in this study. The tannin content not only depends on the plant species provided but also on the specific plants fed, as well as factors such as plant age and growing conditions (reviewed in Tong et al., 2021). The authors recommend this in follow-up studies to better understand the effect of the plant species provided in the food on the development of metabolic disturbances.

## Limitations and further future recommendations

The limited population of rhinoceroses studied here had high levels of TS%, insulin, and inflammatory markers, making the identification of associations difficult. While TS% appears to be a highly sensitive indicator of iron accumulation in black rhinoceroses, it should be noted that it cannot be used as the sole indicator for diagnosing IOD in this species (Sullivan et al., 2020).

In humans, it has been demonstrated that inflammation and obesity can lead to insulin resistance, resulting in dysmetabolic Fe overload (Barbalho et al., 2023). This phenomenon has also been hypothesized in black rhinoceroses by Schook et al. (2015); but see Clauss and Hatt (2006). Insulin resistance may further cause hypophosphatemia (reviewed in Gaasbeek and Meinders, 2005; Haap et al., 2006). Hypophosphatemia is also observed in *ex-situ* rhinoceroses (Dennis et al., 2007; Radeke-Auer et al., 2023). Phosphate levels (and Ca) were not analyzed in this study. However, in other species, such as alpacas, hypovitaminosis D_3_ has been associated with hypophosphatemia (Van Saun et al., 1996). This factor should, therefore, be considered in further research. Stress markers, such as glucocorticosteroids, were also not analyzed in this study, but stress impacts P and metal metabolism in other species (Stanton and Koeppen, 2004; Spiers et al., 2022). It is, therefore, recommended that in addition to P levels, glucocorticosteroids or other indicators of stress be analyzed in future research. A recent study on pathology in European black rhinoceroses suggested that chronic stress could contribute to the development of IOD (Radeke-Auer et al., 2023). In addition to nutrition and stress, exercise may play an important role in the development of metabolic disorders in black rhinoceroses. In captivity, black rhinoceroses appear to be less active (Dierenfeld, 1997). A pilot study involving a limited number of *ex-situ* black rhinoceroses revealed that increased activity led to a significant decrease in inflammatory factors and Fe overload (Bryk, 2009).

## Conclusion

The studied population of *ex-situ* rhinoceroses exhibited high serum TS%, insulin, and inflammatory markers (SAA, IL-6, TNF-α and IFN-γ), which are consistent with those of other studies with ex-situ rhinoceroses. Moreover, 25-hydroxyvitamin D levels were significantly lower in winter versus summer, which is in line with previous studies. No correlations were found between TS%, inflammatory markers, 25(OH)D, and insulin levels.

The microbiome did not significantly differ among institutions; however, some bacteria were present at higher levels alongside a one-time spike in inflammatory markers. Overall, Firmicutes were identified as the most abundant phylum, followed by Bacteroidetes, which is consistent with the majority of existing studies. This may also explain why the butyrate levels are notably high compared to other studies performed on wild black rhinoceroses. No correlations between the SFCAs and medium-chain fatty acids with the serum parameters were found. The present study demonstrates that, while most bacteria produce SCFAs, this is not universally applicable. This finding represents a refinement of the conventional bulk SCFA analysis of feces through functional analysis. In contrast to the microbiome, the mycobiome showed significant differences among institutions. In addition, this study revealed that all identified gut-associated fungi are involved in cellulose degradation, a process also undertaken by some of the bacteria present. This aligns with the dietary habits of the black rhinoceros. The differential abundance analysis of plant species revealed that the abundance of plant types in the diet might impact total solids percentage (TS%), possibly through iron binding or enhancing iron absorption. Future research on IOD based on diet might reveal a dietary composition associated with low TS% only.

## Data Availability

The 16S rRNA, ITS2 and matK data sets generated in this study were deposited in the European Nucleotide Archive (ENA) under the project accession number PRJEB80731.
