# Correction: Rhinoceromics: a multi-amplicon study with clinical markers to transferrin saturation levels in ex-situ black rhinoceros (*Diceros bicornis michaeli*)

**DOI:** 10.3389/fmicb.2025.1644681

**Published:** 2025-06-27

**Authors:** Linda G. R. Bruins-van Sonsbeek, Martie C. M. Verschuren, Sonja Kaal, Peter W. Lindenburg, Kees (C.) W. Rodenburg, Marcus Clauss, Arjen G. C. L. Speksnijder, Victor P.M.G. Rutten, Bas F. J. Bonnet, Floyd Wittink

**Affiliations:** ^1^Veterinary Department, Rotterdam Blijdorp Zoo, Rotterdam, Netherlands; ^2^Avans University of Applied Sciences, Breda, North Brabant, Netherlands; ^3^LCAB, Department of Analytical BioSciences, University of Applied Sciences Leiden, Leiden, Netherlands; ^4^Clinic for Zoo Animals, Exotic Pets and Wildlife, University of Zurich, Zurich, Switzerland; ^5^LCAB, Department of Environmental Metagenomics, University of Applied Sciences Leiden, Leiden, Netherlands; ^6^Department Understanding Evolution, Naturalis Biodiversity Center, Leiden, Netherlands; ^7^Section Immunology, Div Infectious Diseases and Immunology, Dept Biomolecular Health Sciences|Faculty of Veterinary Medicine, Utrecht University, Utrecht, Netherlands; ^8^Department of Veterinary Tropical Diseases, Faculty of Veterinary Science, University of Pretoria, Pretoria, South Africa; ^9^LCAB, Department of Bioinformatics, University of Applied Sciences Leiden, Leiden, Netherlands

**Keywords:** *Diceros bicornis michaeli*, black rhinoceros, iron overload disorder, short- and medium-chain fatty acid analysis, microbiome, vitamin D, inflammatory markers, mycobiome

There was a mistake in the caption of Figures 3, 4, 7, and 8 as published. The captions of these figures have been swapped. The corrected caption of figure/table [insert figure/table number appears below.

Figure 3. Microbial α-diversity according to grouped rhino_name data. **(A)** Observed diversity **(B)**, Chao1 diversity **(C)**, Shannon diversity, and **(D)** Inverse Simpson. All data have been tested for significant differences by a Kruskal-Wallis test at *p* = 0.05.

Figure 4. Principal components of rhino fecal samples analyzed by 16S for selected distance matrices. **(A)** Aitchison distance and **(B)** Bray-Curtis dissimilarity.

Figure 7. Significant abundance of plant species (matK, FDR < 0.05) between rhinoceroses with low TS% (<63.8%). The threshold of 63.8% was taken from the median TS% of the population under study. The significant abundances are visualized as a Volcanoplot. The orange dot represents significant abundance, and the green dot represents both significant and fold changed >2 of plant species concerning TS class.

Figure 8. Transferrin saturation (TS) vs. age in years. The black dots illustrate every unique sample, the black line shows the trend, the red striped line is a TS of 50%, the black striped line is a TS level of ~60%, and the median of the rhinoceroses in the current study.

The original version of this article has been updated.

