# Supplementary figures and images for: Rhinoceromics: a multi-amplicon study with clinical markers to transferrin saturation levels in ex-situ black rhinoceros (Diceros bicornis michaeli)
Source: Front Microbiol. 2025 May 29;16:1515939. doi: 10.3389/fmicb.2025.1515939 (PMC12158930; doi:10.3389/fmicb.2025.1515939)

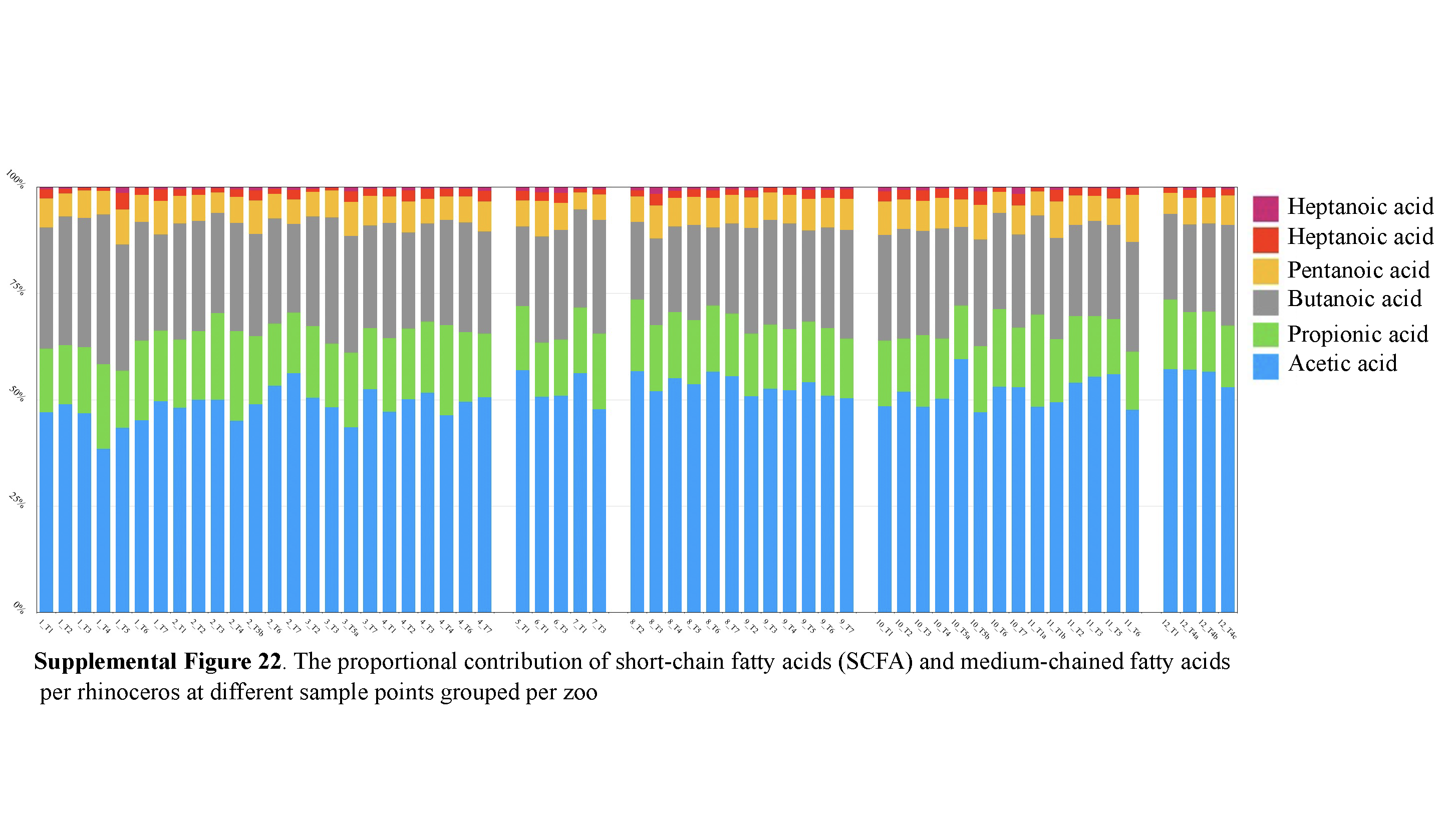

Supplement: Supplementary file 1 [file Data_Sheet_1.zip › supplementary data 1515939/supplement_Figure22.tiff]

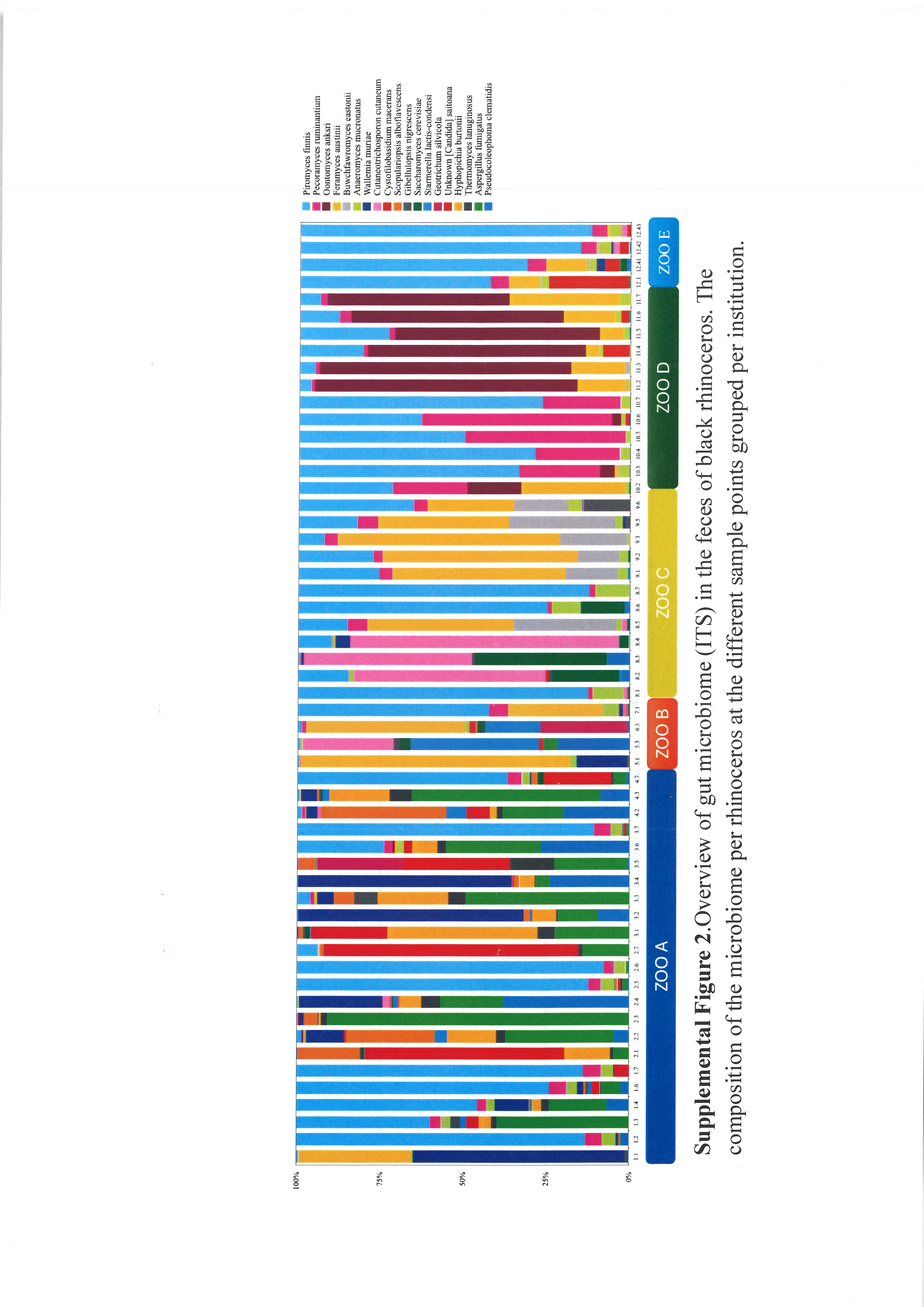

Supplement: Supplementary file 1 [file Data_Sheet_1.zip › supplementary data 1515939/supplement_Figure02.tiff]

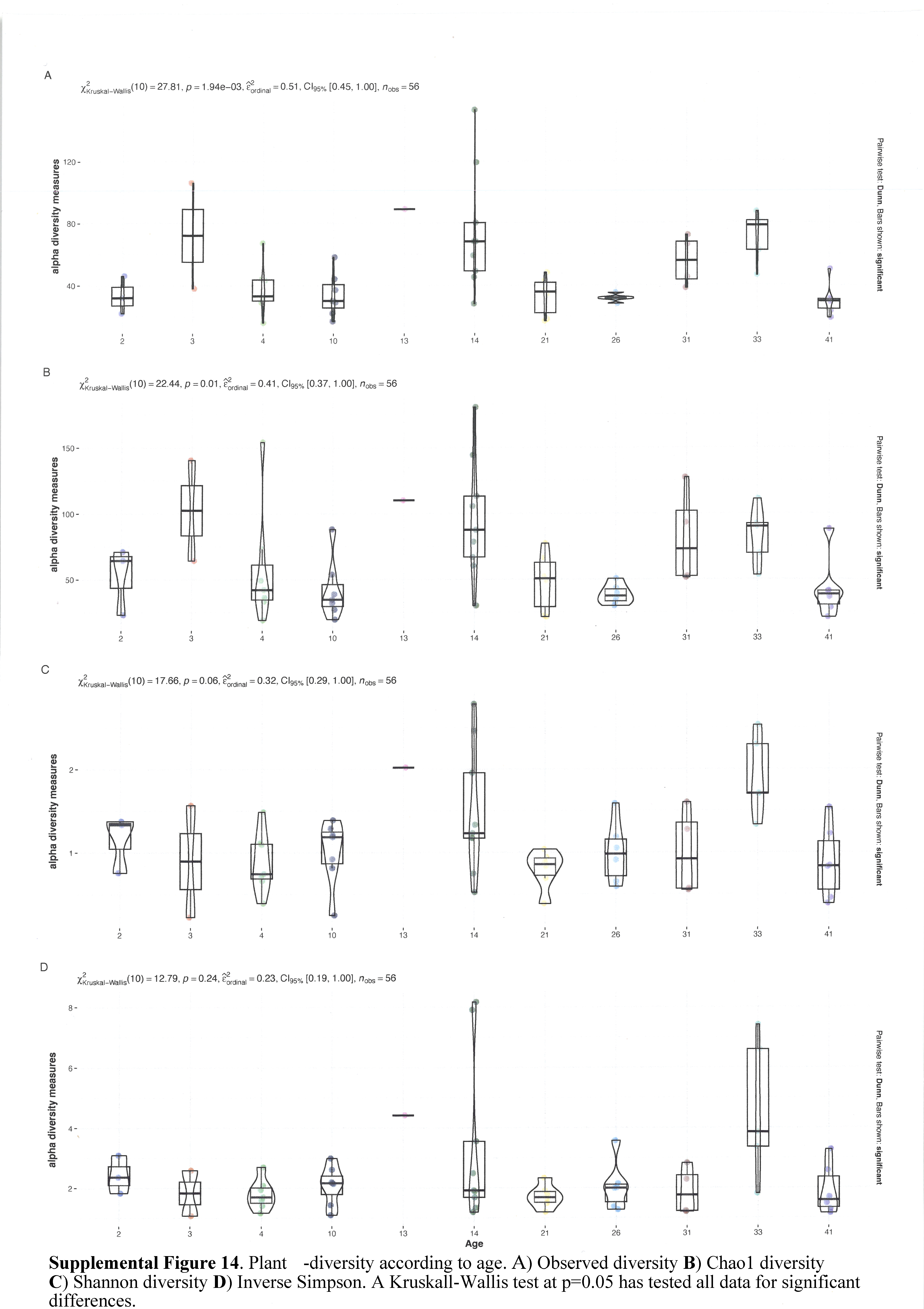

Supplement: Supplementary file 1 [file Data_Sheet_1.zip › supplementary data 1515939/supplement_Figure14.tiff]

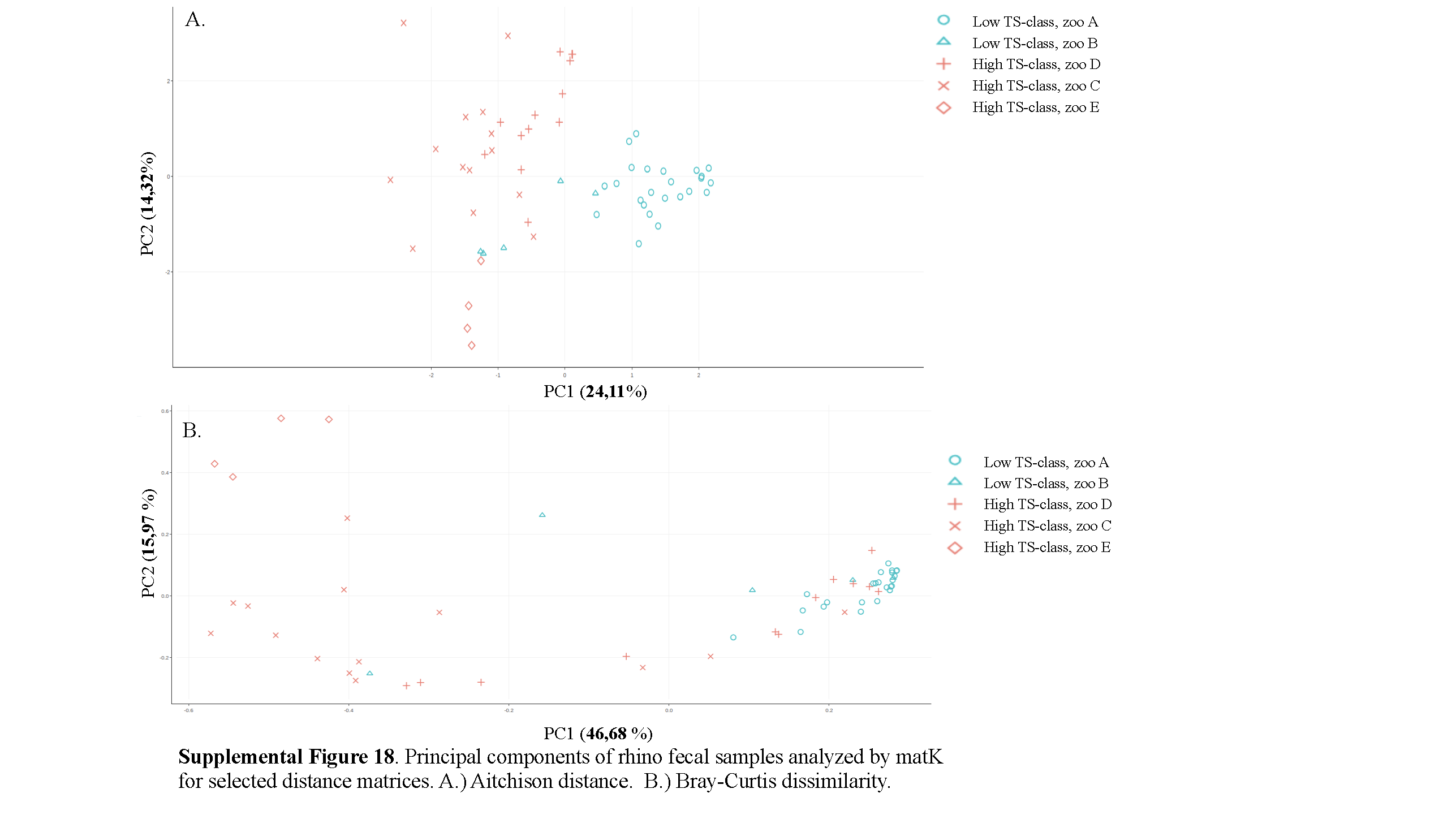

Supplement: Supplementary file 1 [file Data_Sheet_1.zip › supplementary data 1515939/supplement_Figure_18 18-2.tiff]

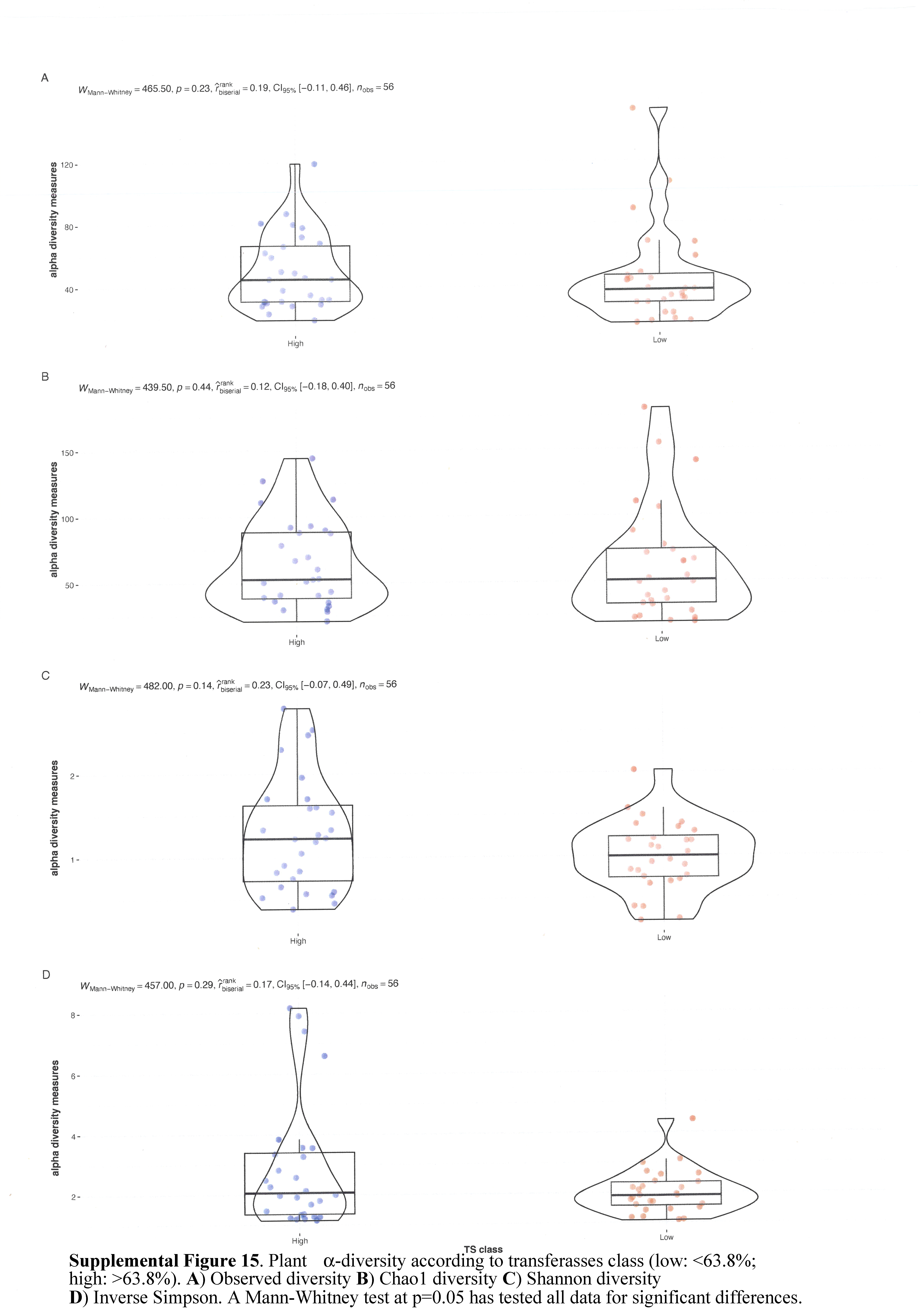

Supplement: Supplementary file 1 [file Data_Sheet_1.zip › supplementary data 1515939/supplement_Figure15.tiff]

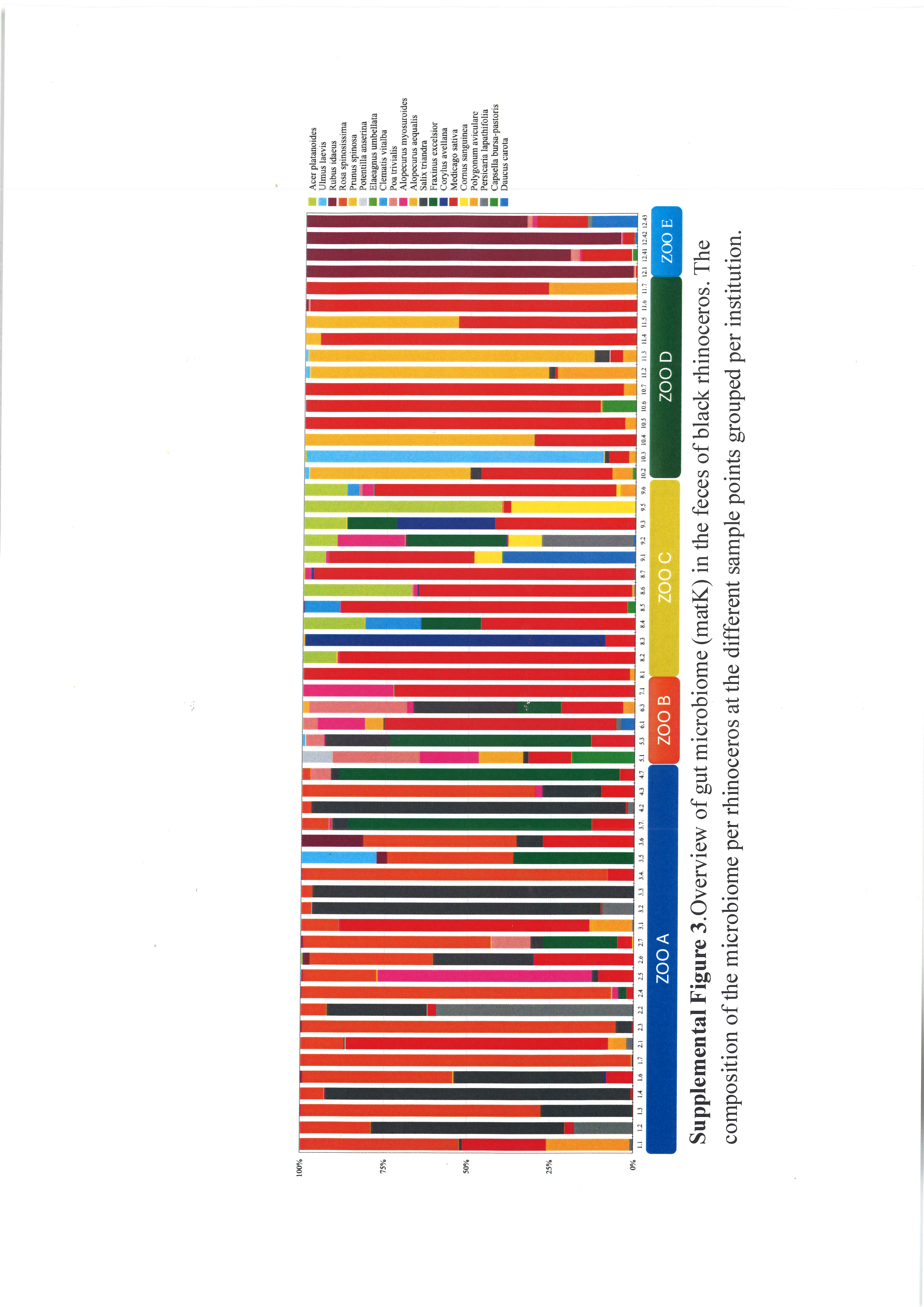

Supplement: Supplementary file 1 [file Data_Sheet_1.zip › supplementary data 1515939/supplement_Figure03.tiff]

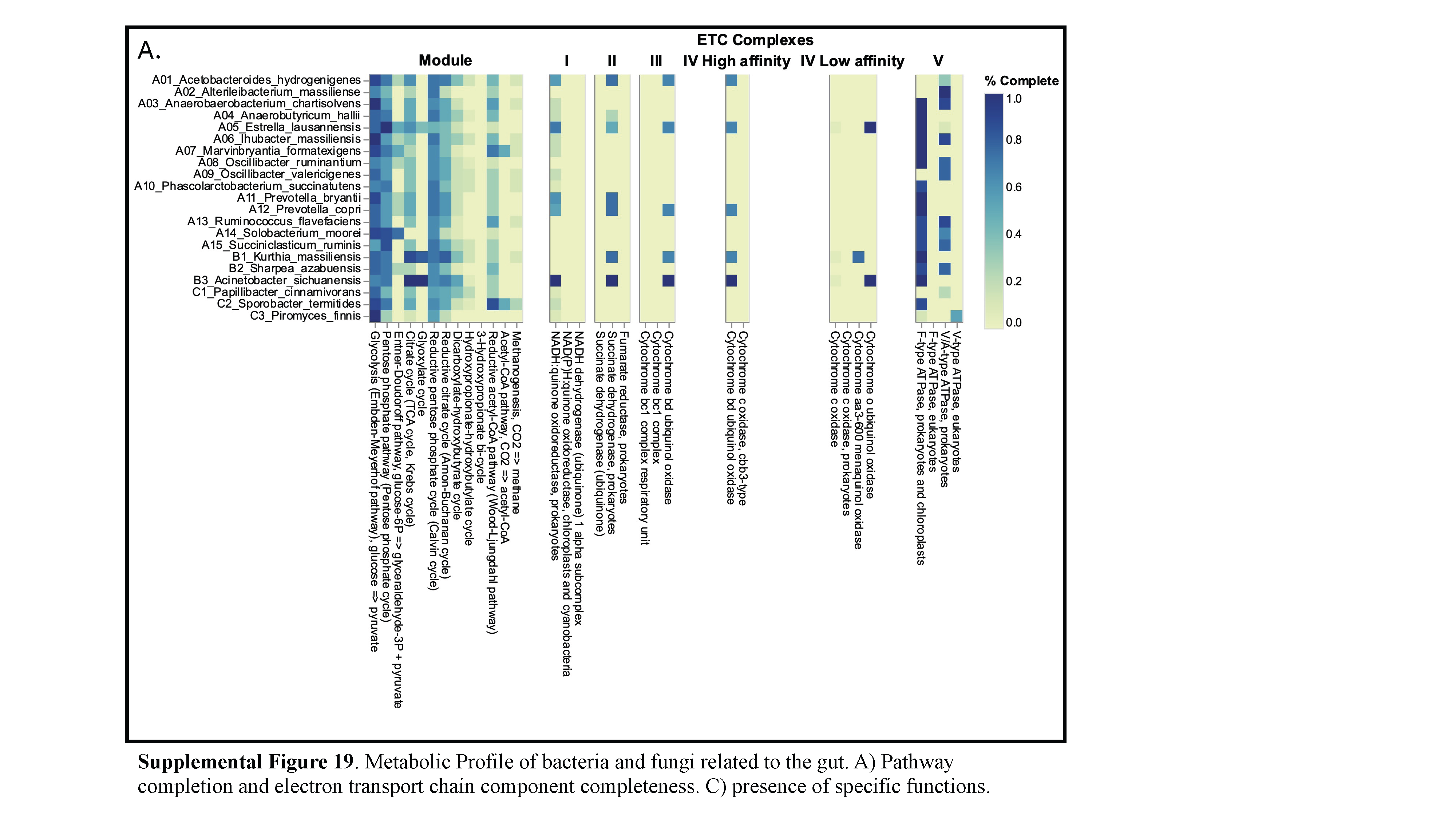

Supplement: Supplementary file 1 [file Data_Sheet_1.zip › supplementary data 1515939/supplement_Figure19.tiff]

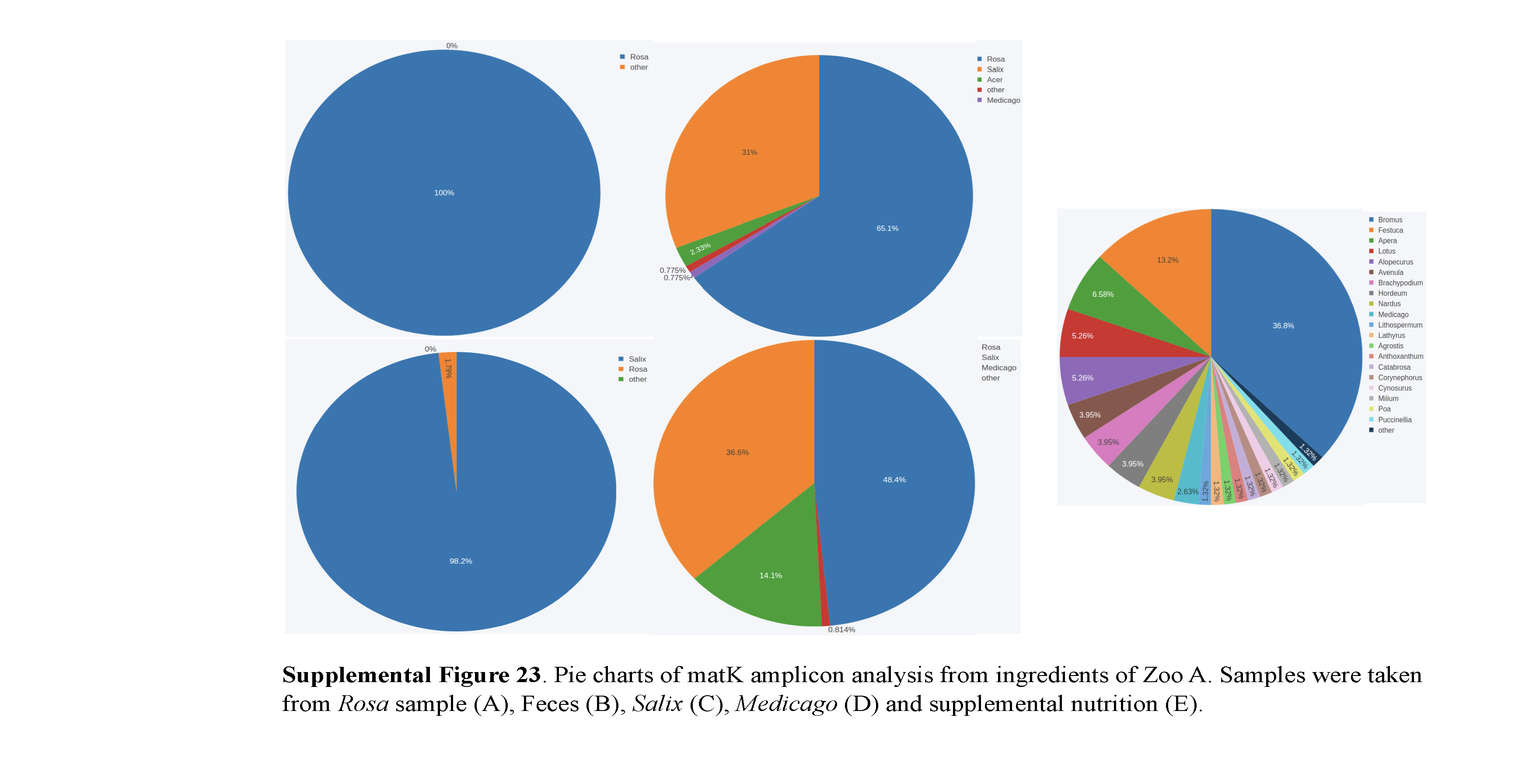

Supplement: Supplementary file 1 [file Data_Sheet_1.zip › supplementary data 1515939/supplement_Figure23.tiff]

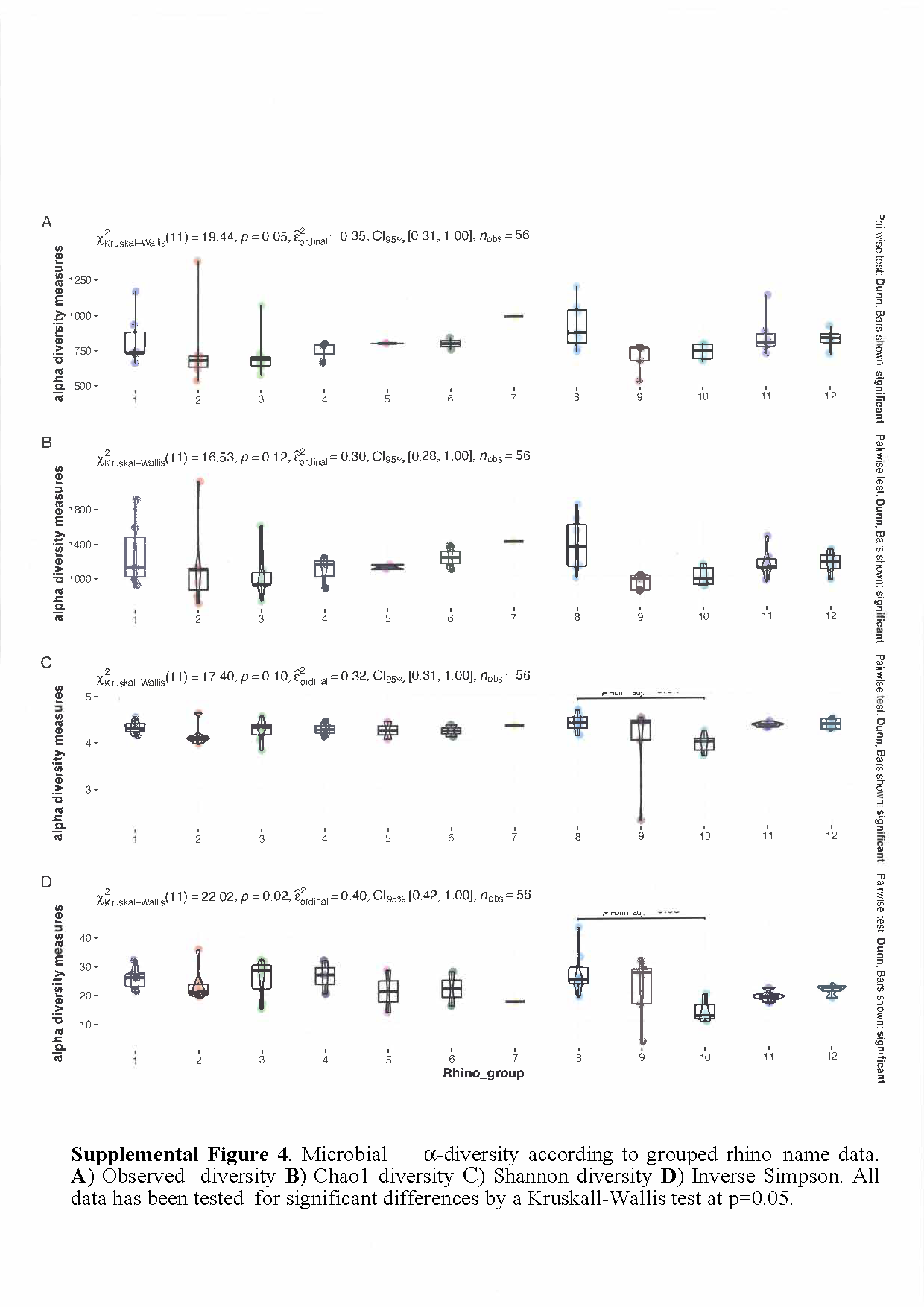

Supplement: Supplementary file 1 [file Data_Sheet_1.zip › supplementary data 1515939/supplement_Figure04.tiff]

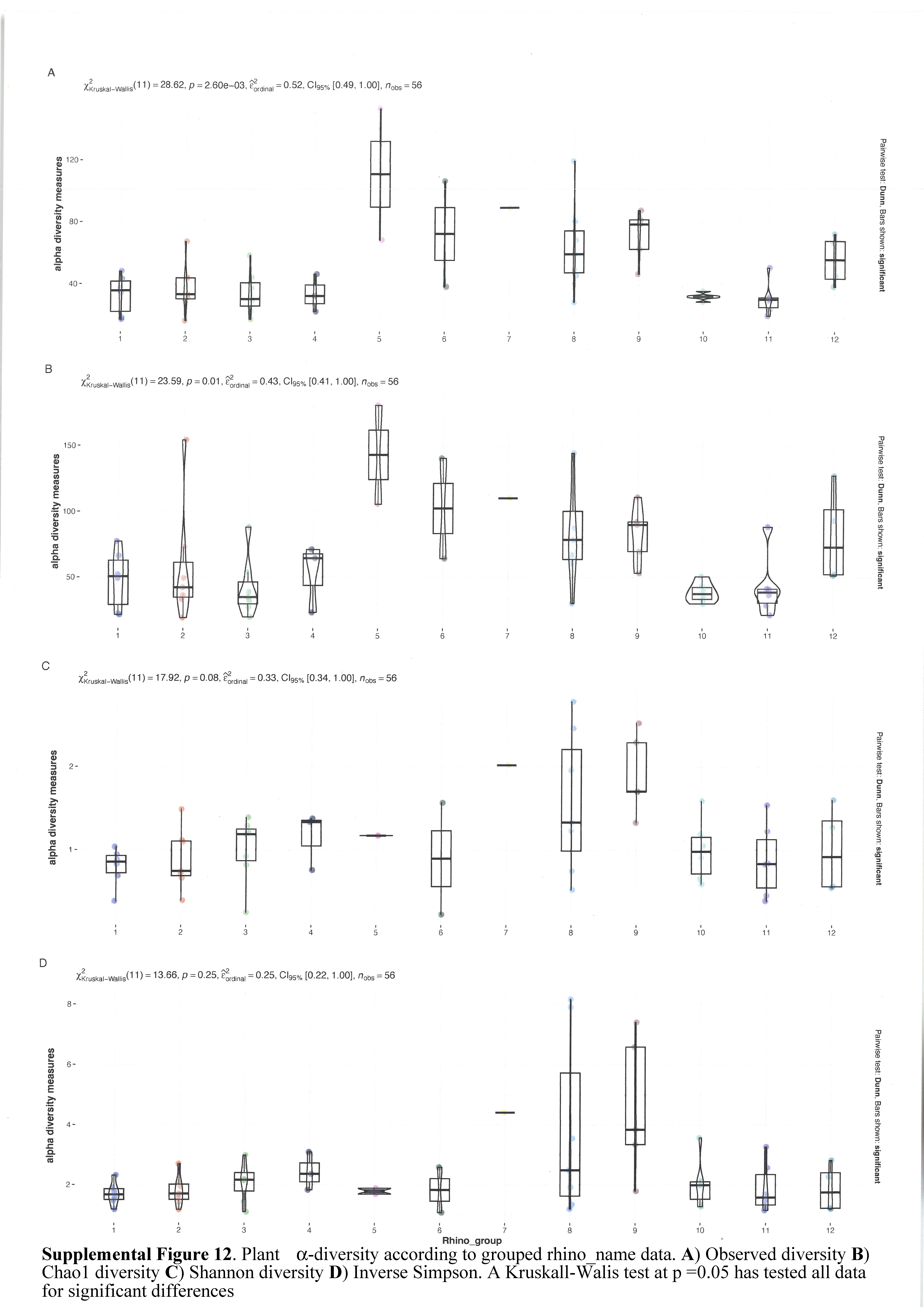

Supplement: Supplementary file 1 [file Data_Sheet_1.zip › supplementary data 1515939/supplement_Figure12.tiff]

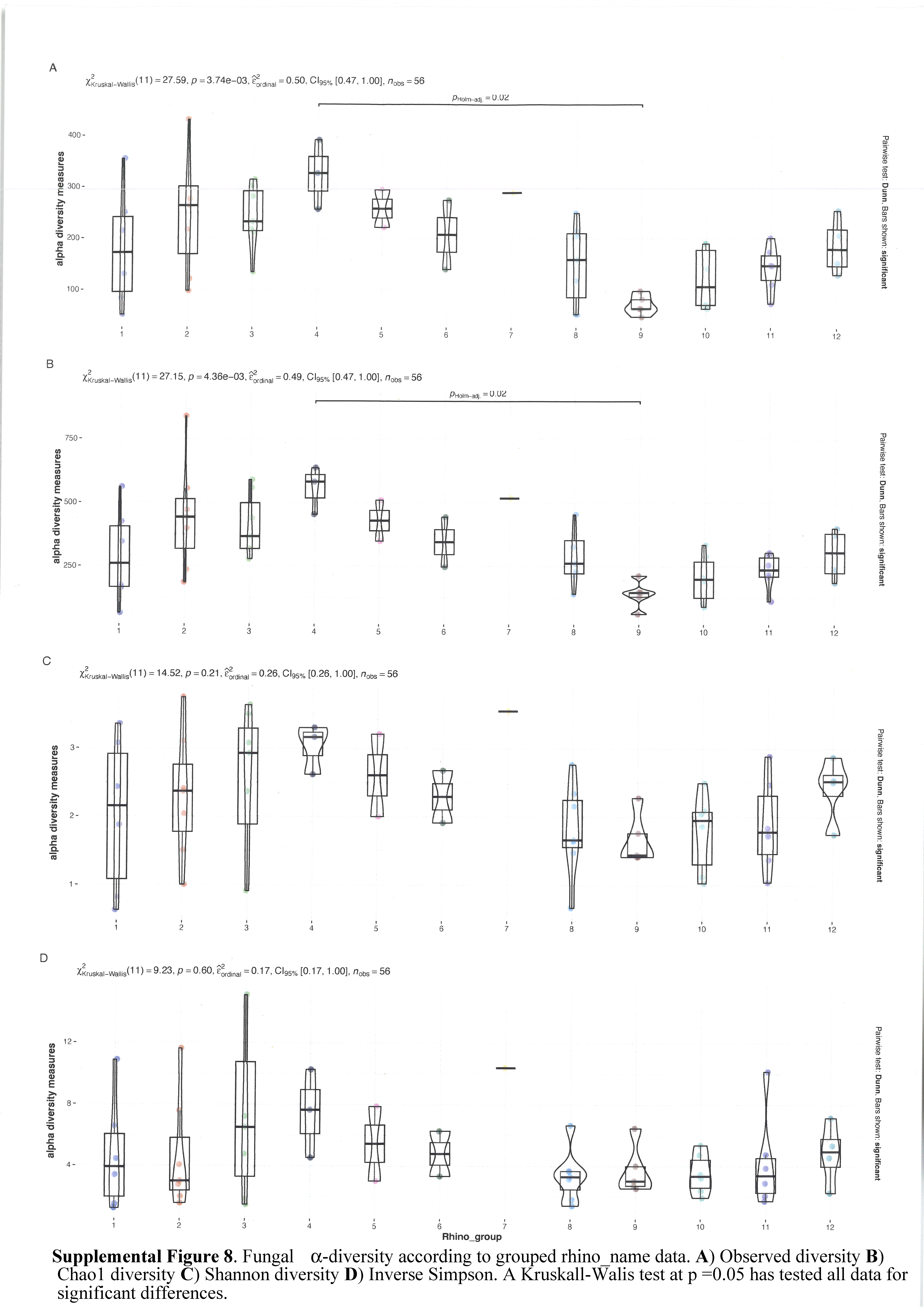

Supplement: Supplementary file 1 [file Data_Sheet_1.zip › supplementary data 1515939/supplement_Figure08.tiff]

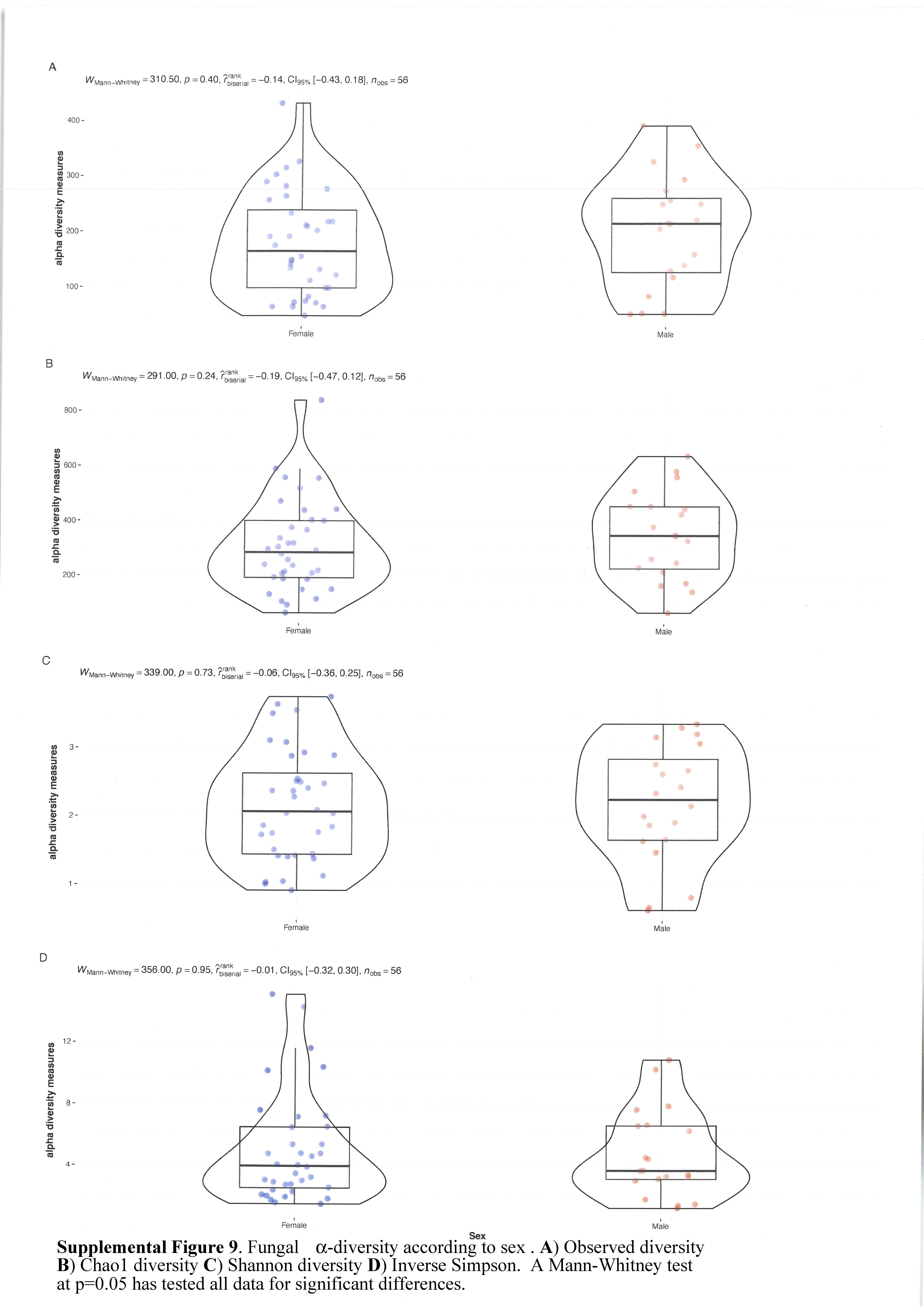

Supplement: Supplementary file 1 [file Data_Sheet_1.zip › supplementary data 1515939/supplement_Figure09.tiff]

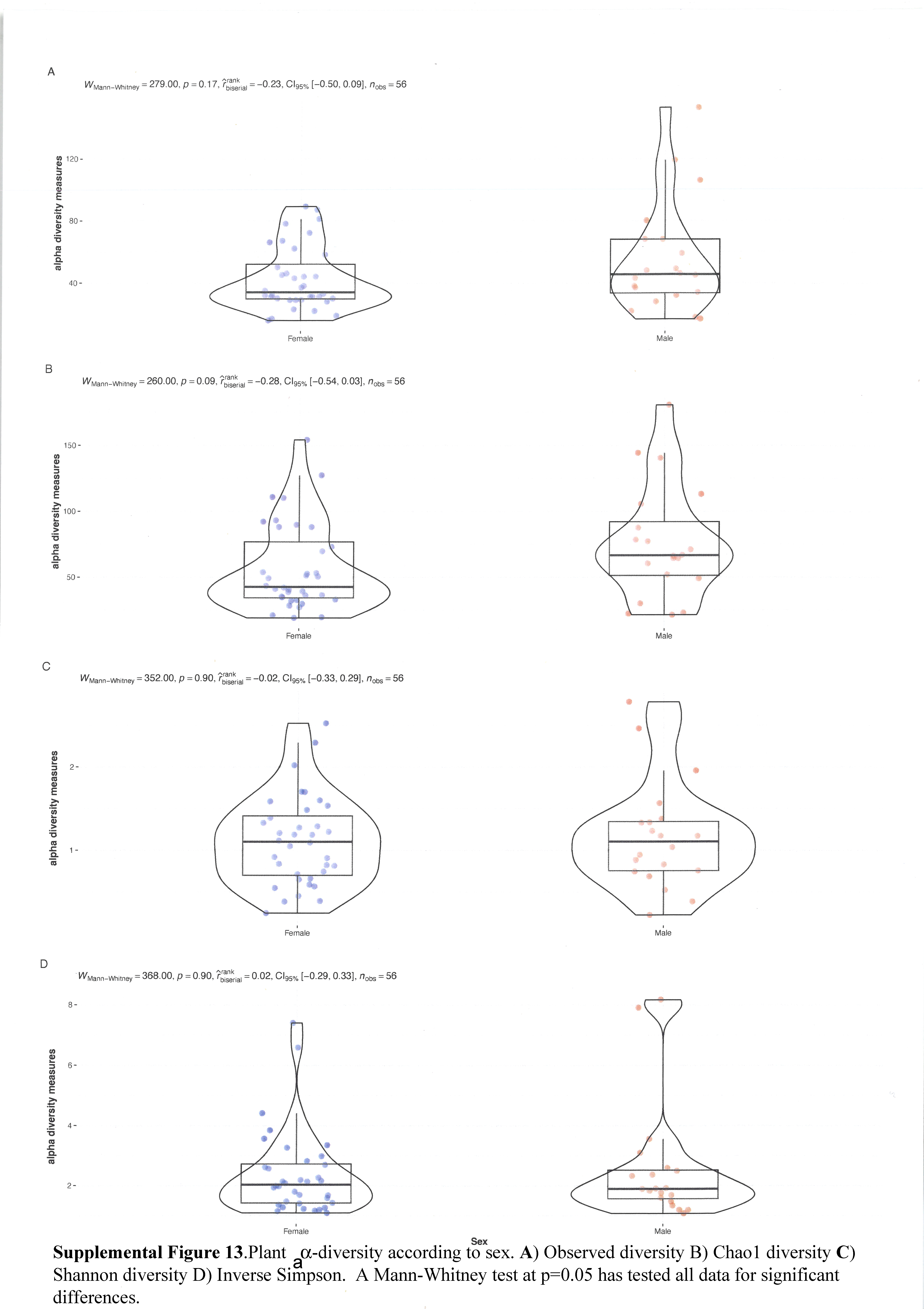

Supplement: Supplementary file 1 [file Data_Sheet_1.zip › supplementary data 1515939/supplement_Figure13.tiff]

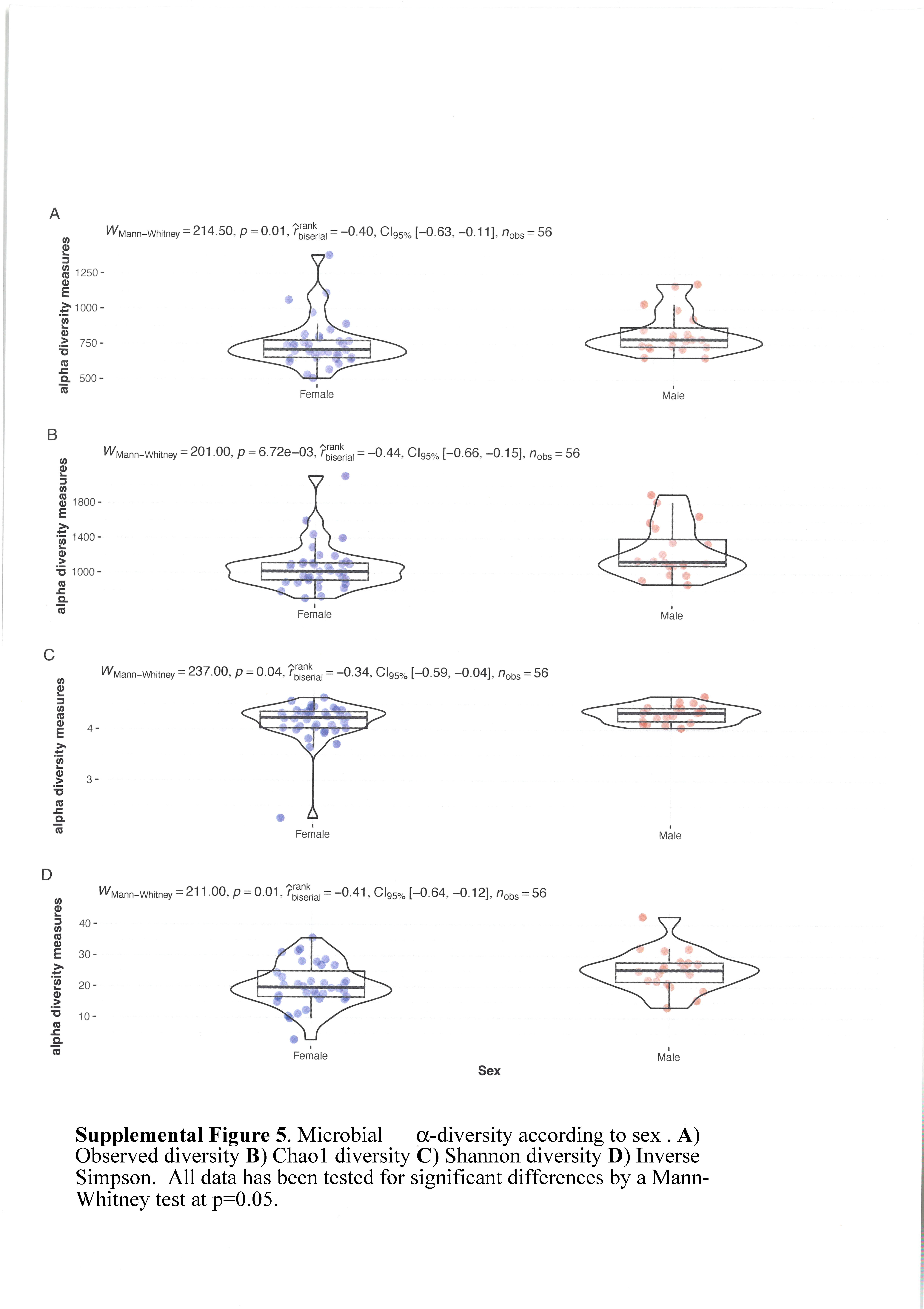

Supplement: Supplementary file 1 [file Data_Sheet_1.zip › supplementary data 1515939/supplement_Figure05.tiff]

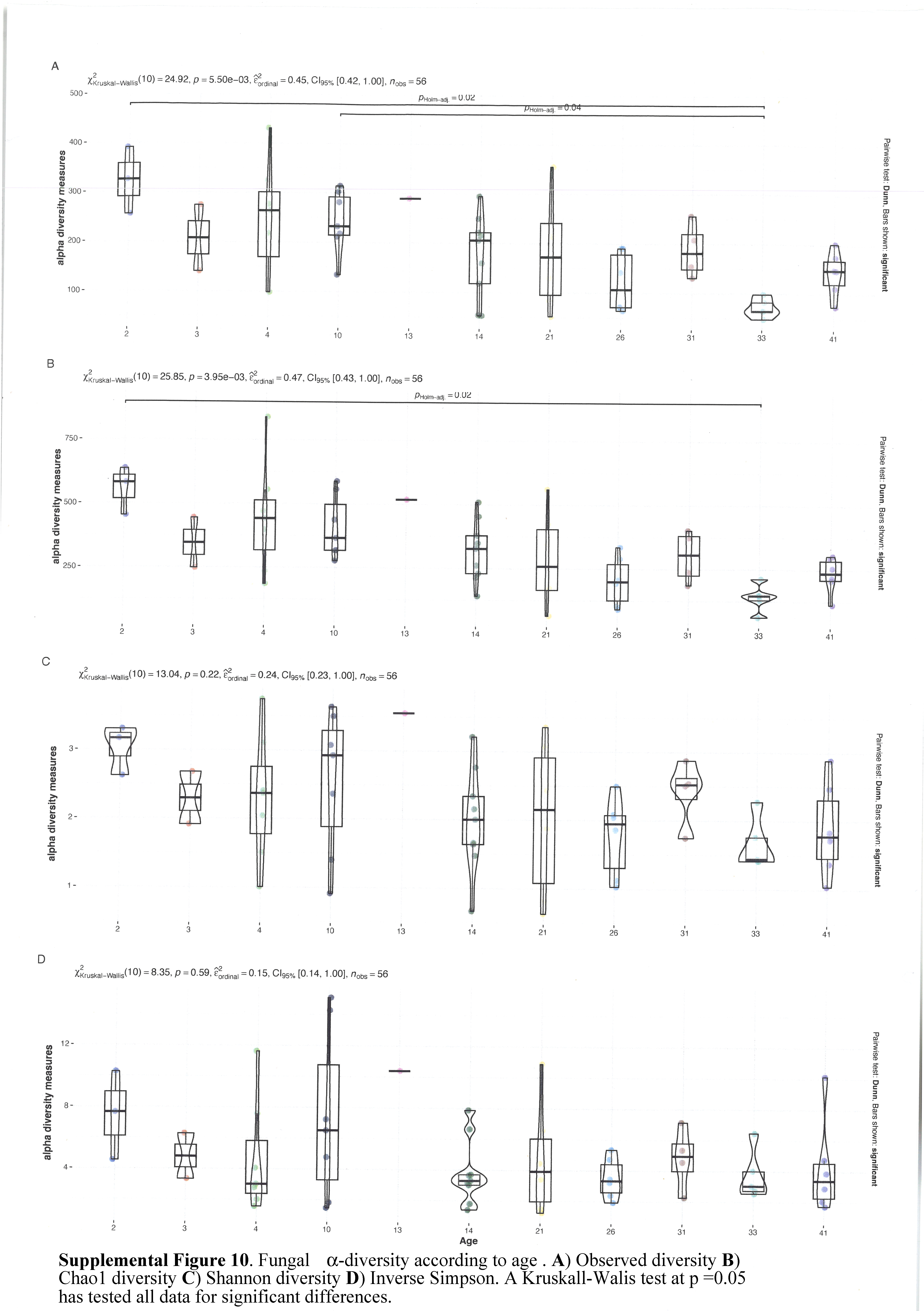

Supplement: Supplementary file 1 [file Data_Sheet_1.zip › supplementary data 1515939/supplement_Figure10.tiff]

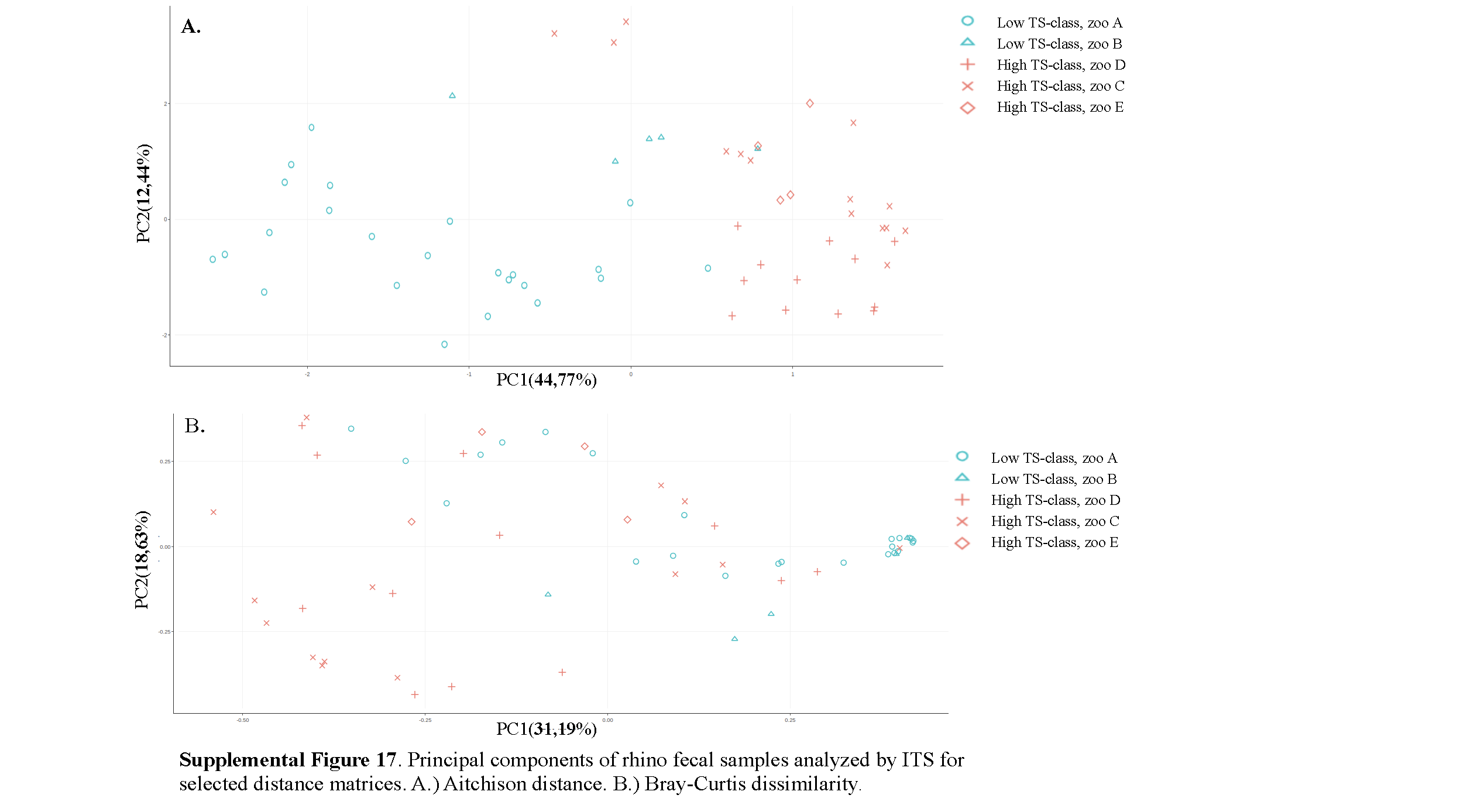

Supplement: Supplementary file 1 [file Data_Sheet_1.zip › supplementary data 1515939/supplement_Figure_17 18-2.tiff]

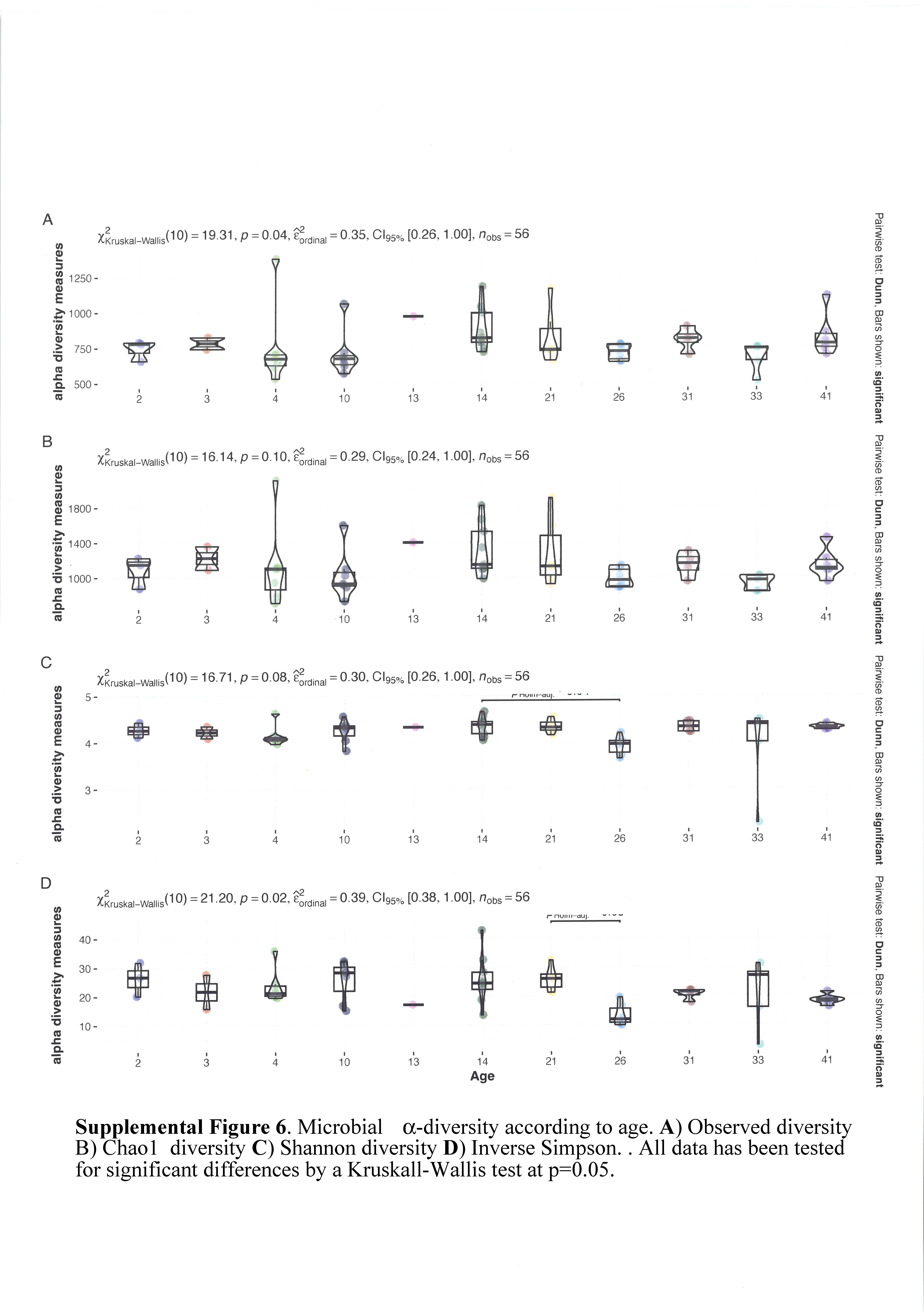

Supplement: Supplementary file 1 [file Data_Sheet_1.zip › supplementary data 1515939/supplement_Figure06.tiff]

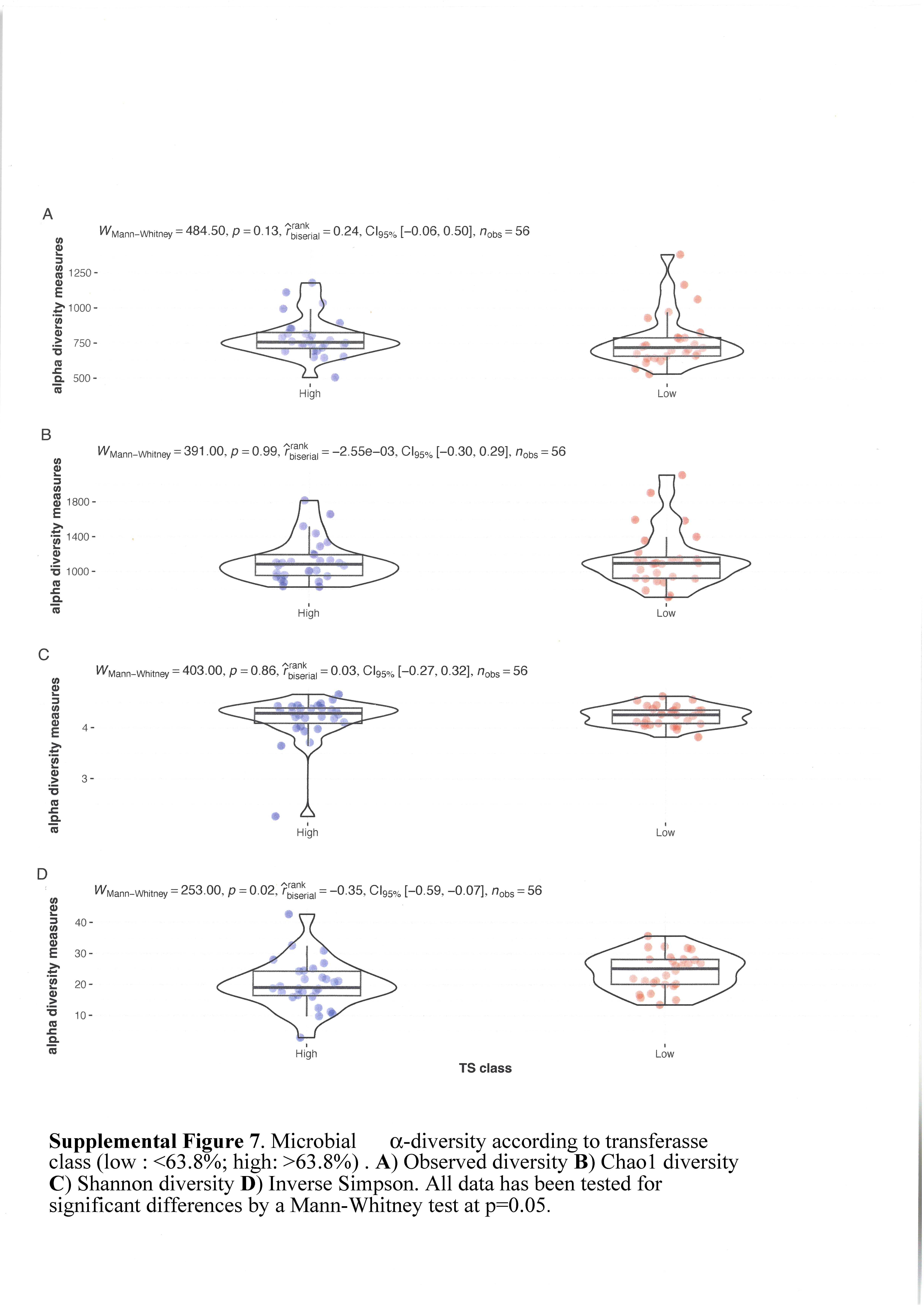

Supplement: Supplementary file 1 [file Data_Sheet_1.zip › supplementary data 1515939/supplement_Figure07.tiff]

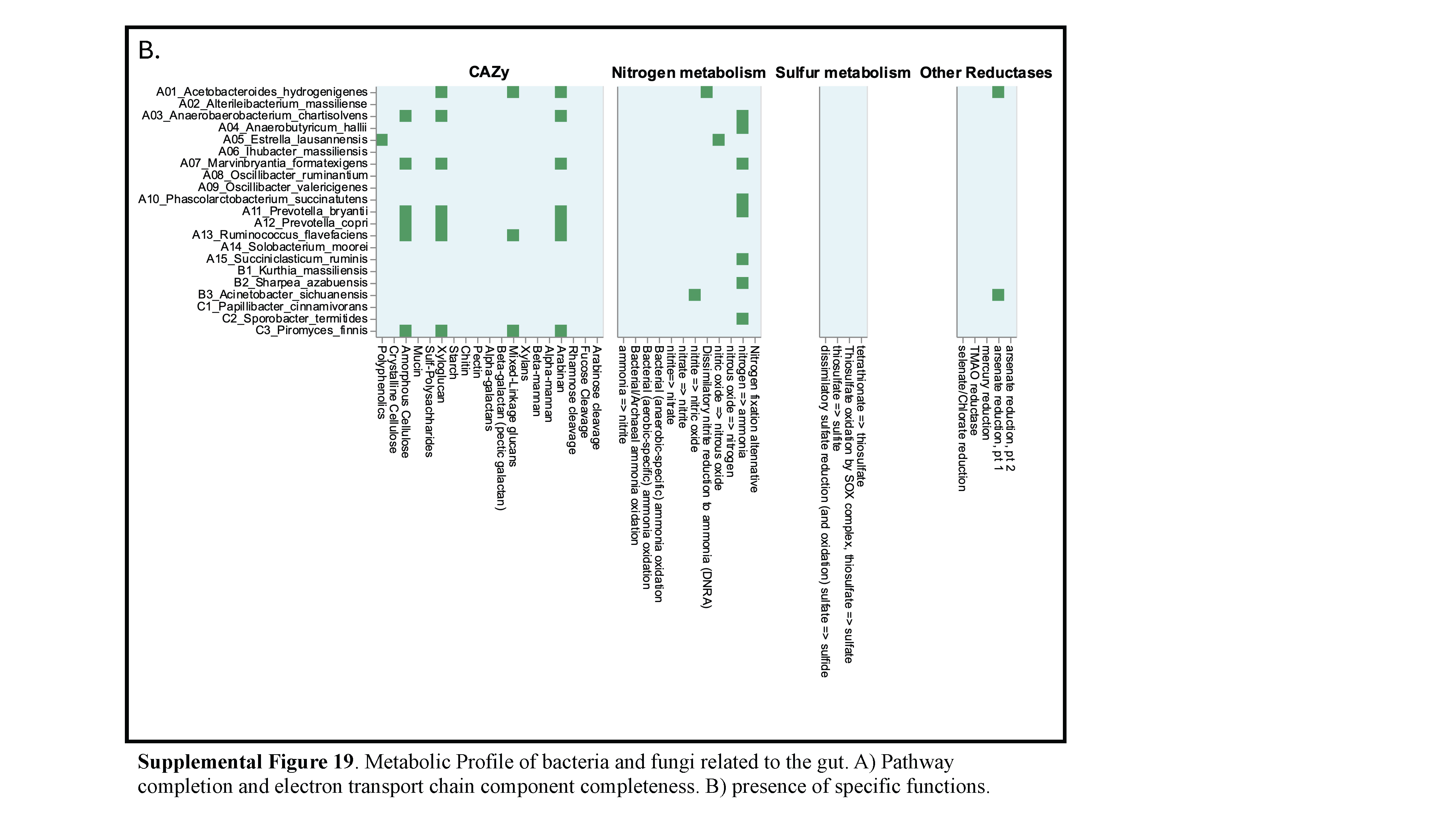

Supplement: Supplementary file 1 [file Data_Sheet_1.zip › supplementary data 1515939/supplement_Figure19B.tiff]

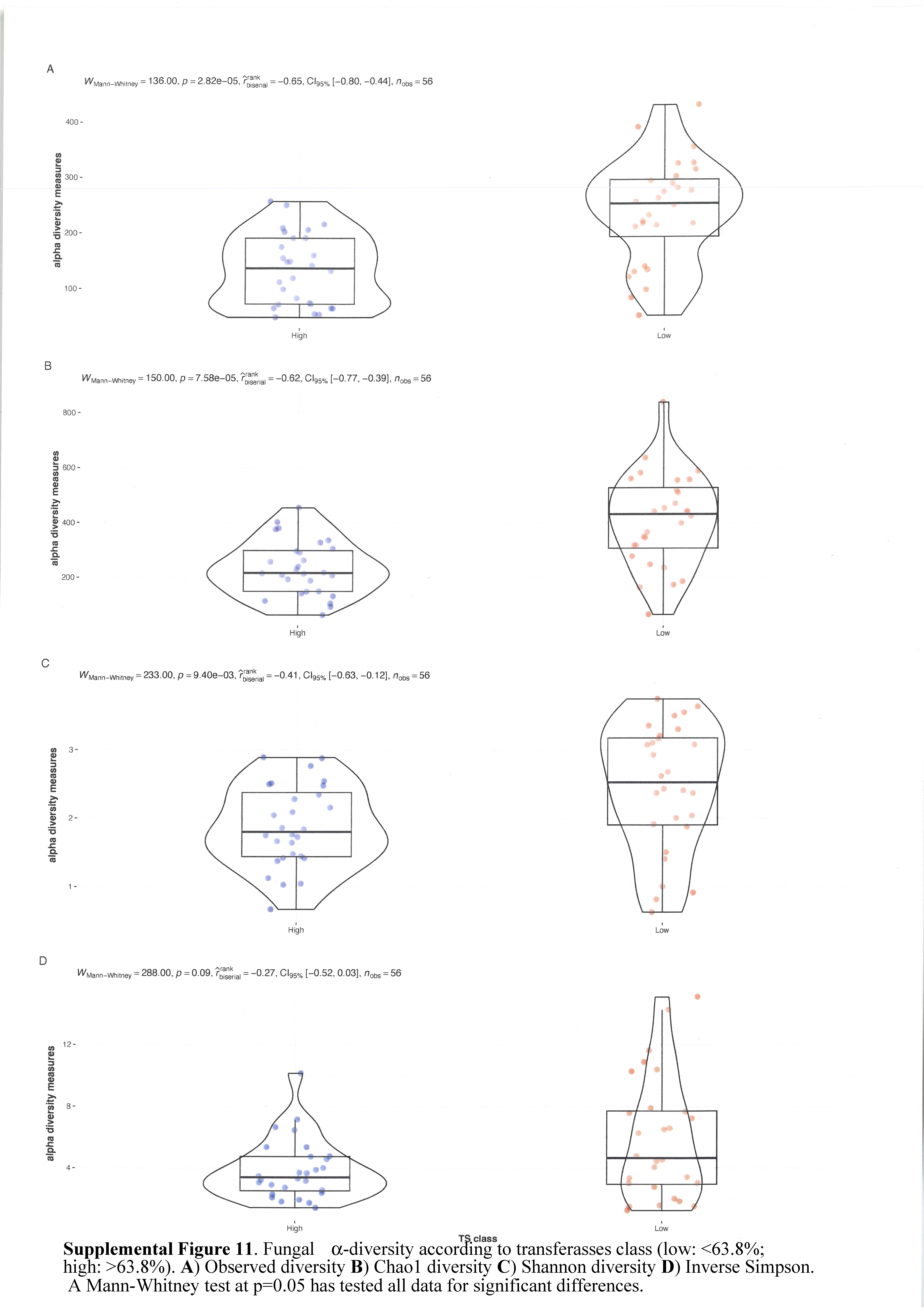

Supplement: Supplementary file 1 [file Data_Sheet_1.zip › supplementary data 1515939/supplement_Figure11.tiff]

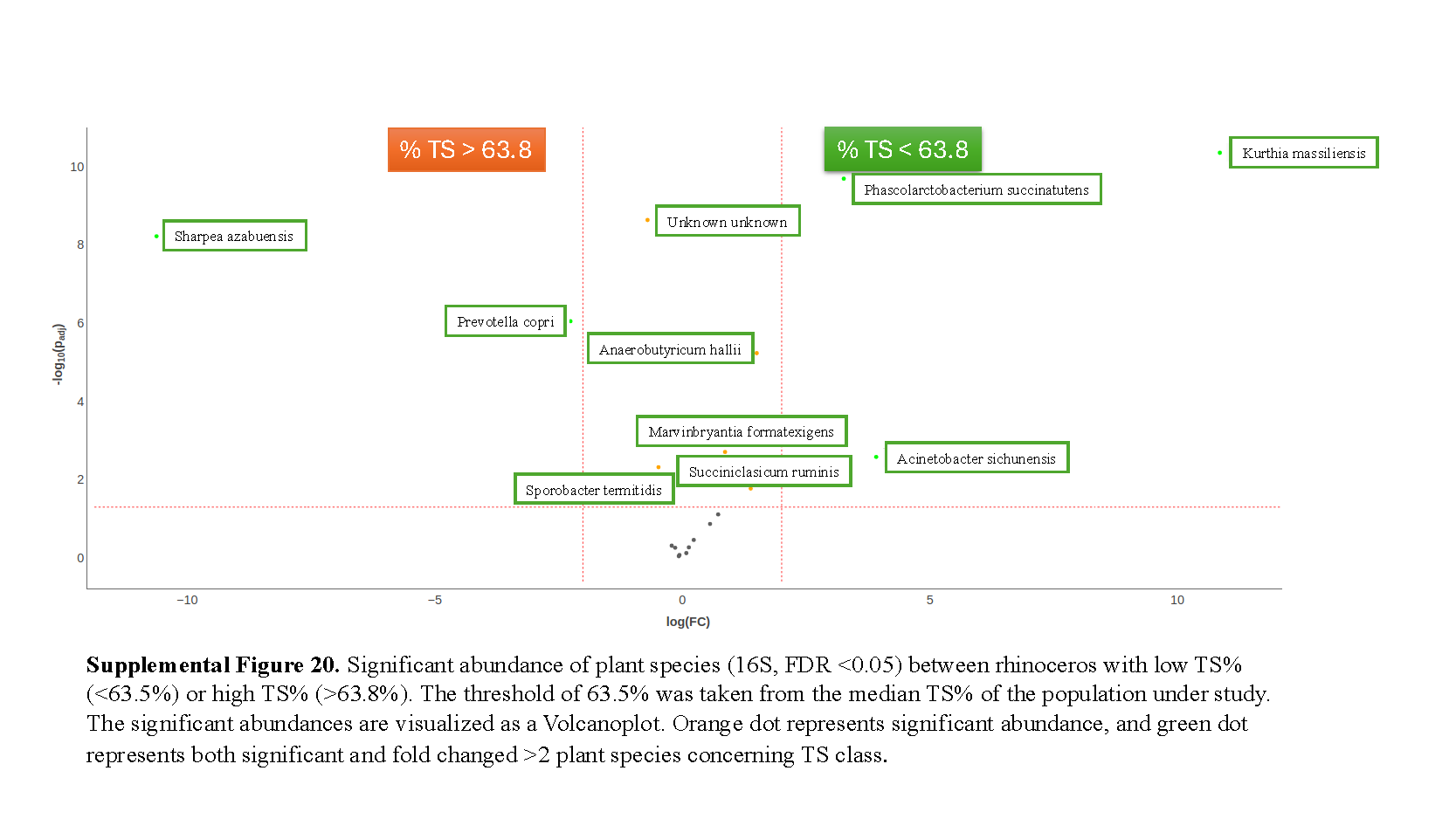

Supplement: Supplementary file 1 [file Data_Sheet_1.zip › supplementary data 1515939/supplement_Figure20.tiff]

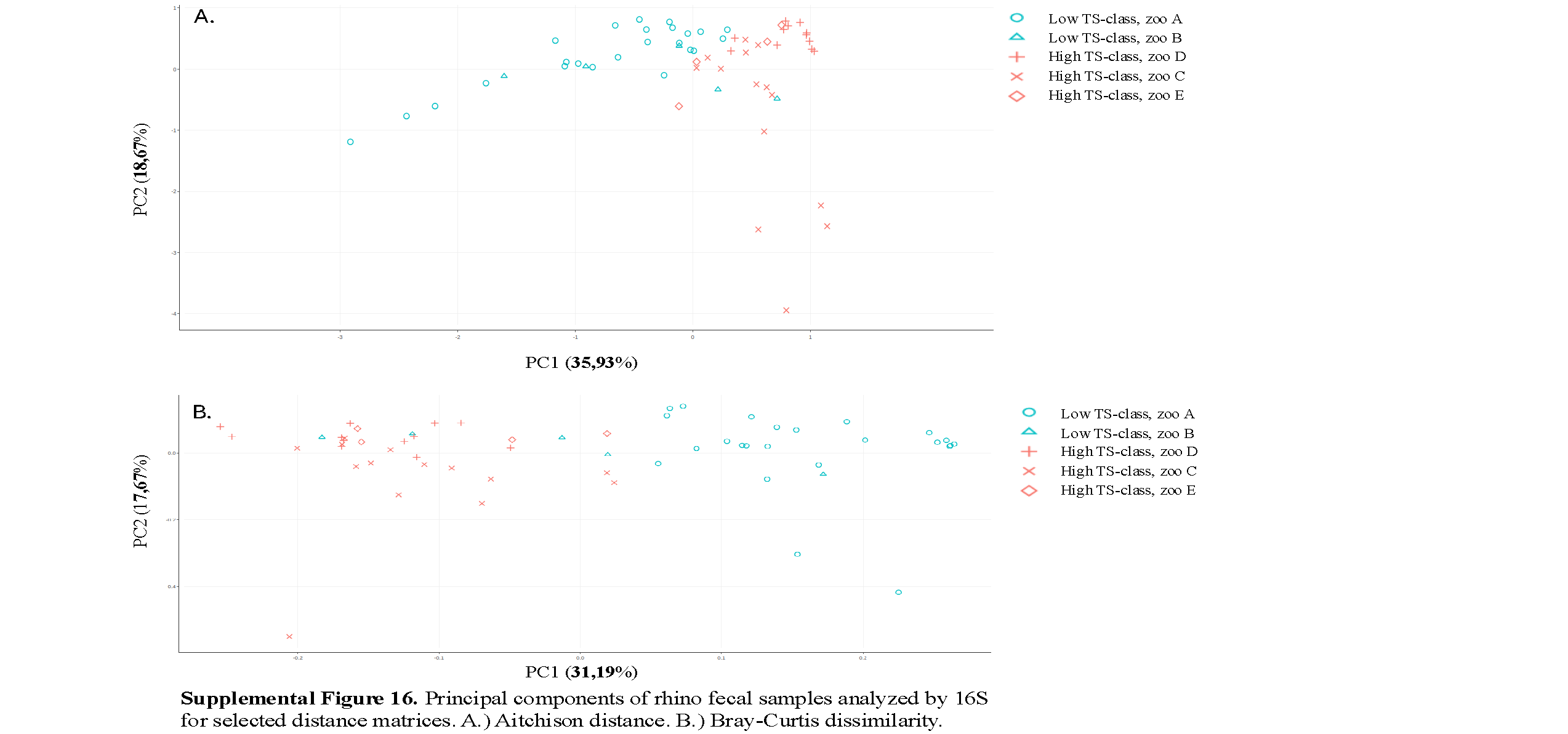

Supplement: Supplementary file 1 [file Data_Sheet_1.zip › supplementary data 1515939/supplement_Figure16.tiff]

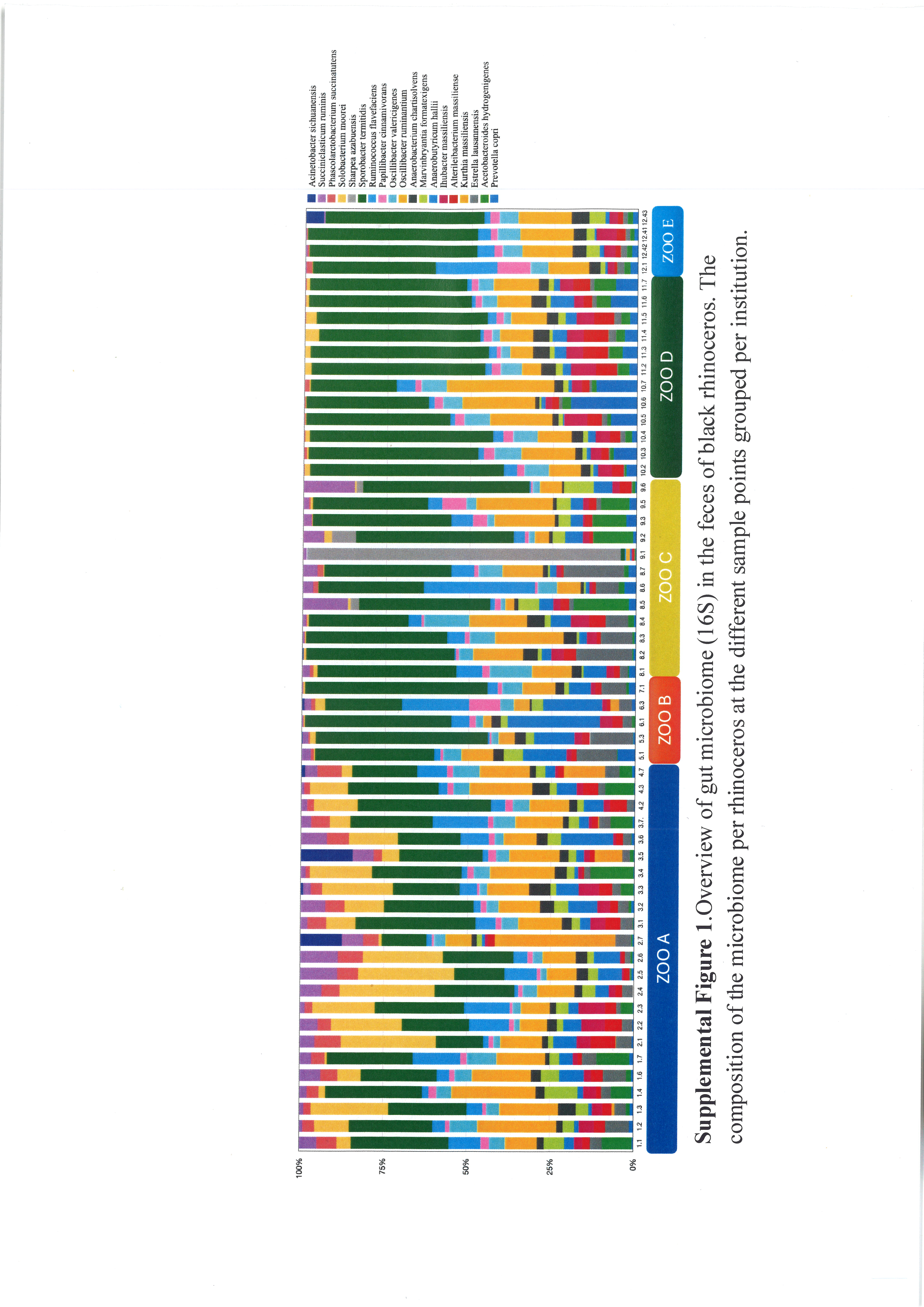

Supplement: Supplementary file 1 [file Data_Sheet_1.zip › supplementary data 1515939/supplement_Figure01.tiff]

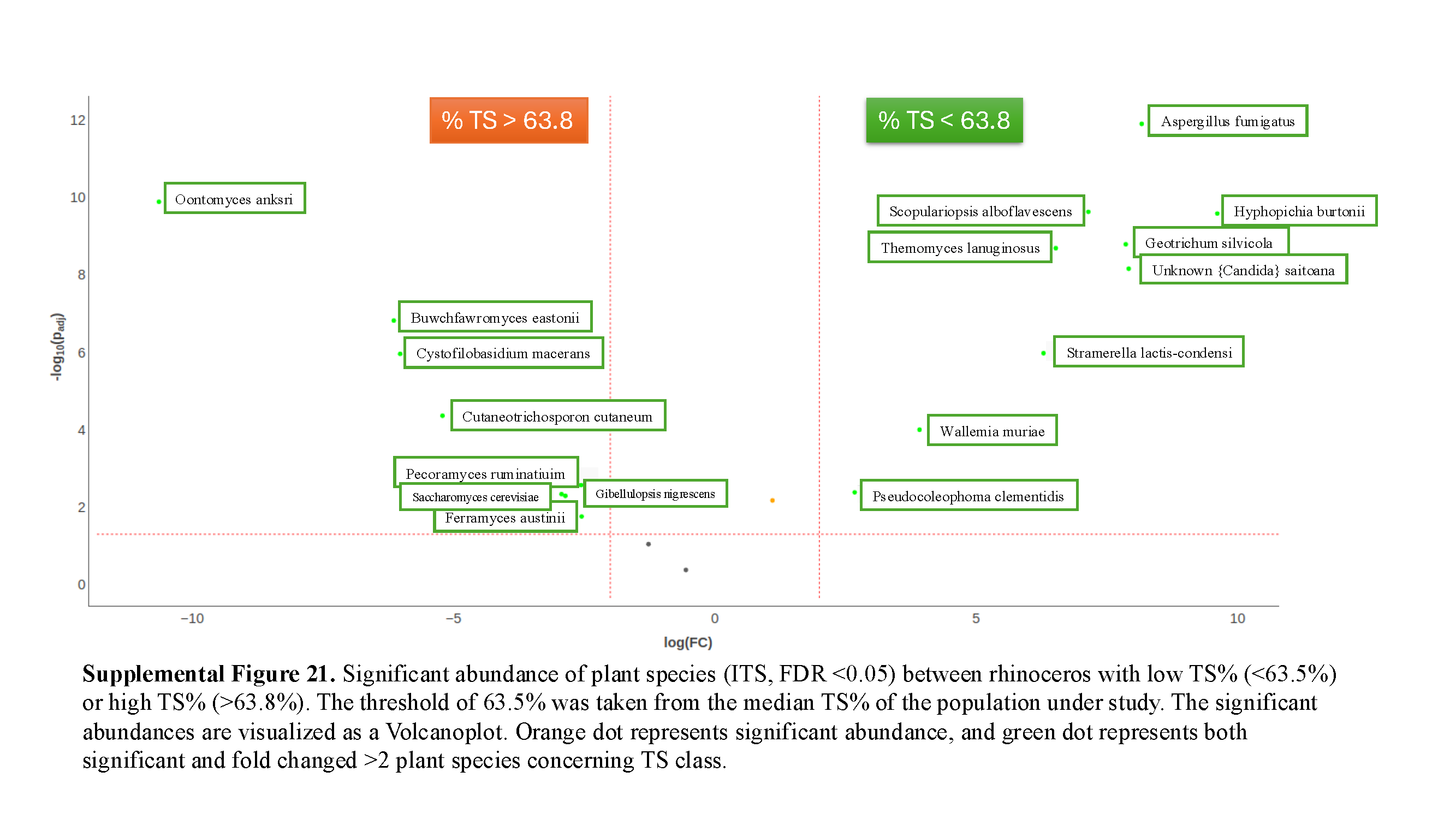

Supplement: Supplementary file 1 [file Data_Sheet_1.zip › supplementary data 1515939/supplement_Figure21.tiff]
